# Understanding Aging Genes and Pathways: Insight from Yeast

**DOI:** 10.3390/cimb48101045

**Published:** 2026-10-09

**Authors:** Giusi Taormina, Mario G. Mirisola

**Affiliations:** 1 Independent Researcher, Palermo 90030, Italy; giusi.taormina86@gmail.com; 2 Department of Stebicef, Università di Palermo, Palermo 90128, Italy

**Keywords:** aging, yeast, model systems, nutrient responsive pathway

## Abstract

Even though there is a general agreement on the heritability of longevity in humans, the complex network of genetic and non-genetic factors affecting our lifespan is far from being fully elucidated. In this context, simple model organisms can still contribute to understanding the fundamental mechanisms involved in longevity, potentially highlighting novel therapeutic targets. Studies in the yeast *Saccharomyces cerevisiae*, the simplest eukaryotic model system, have identified the Ras-cAMP-PKA, TOR and Sch9 pathways as pro-aging signaling cascades. Even though this unicellular fungus is evolutionarily distant from complex mammals, some of the observations made in yeast have been eventually confirmed in higher organisms, including humans. The tractability of this model organism has also enabled a detailed molecular characterization of those pathways identifying potential therapeutic targets. In addition, the molecular characterization of these nutrient-dependent pathways helps screen for molecules capable of modulating stress resistance, cellular nutrient responses and autophagy. This evolutionary conservation, spanning from fission yeast to mammals, positions this model organism as a valuable tool for screening molecules, drugs, and dietary interventions that affect longevity. Here, we describe the principal methodologies used to study aging in yeast, the major findings obtained and their translational validity in higher organisms.

## 1. Introduction

While it is known that yeast has been used for bread, wine and beer making since ancient Egypt, archeological evidence in China dates its usage back to 7000 BCE [1]. In modern times, yeast has established itself as a cornerstone in cellular biology, genetics and molecular biology while remaining essential for the industrial production of food, beverages and, more recently, pharmaceuticals. The reasons for this are mainly its low cost, the high availability of bioinformatic resources, the development of a large array of biochemical and genetic methods and its compatibility with high-throughput technologies. The ascomycete budding and fission yeasts have thus served as important model organisms. Many research fields, including aging [2], have taken advantage of these experimental systems, although their ability to shed light on human longevity has some obvious limitations. Lifespan curves of this unicellular organism, in fact, cannot provide information on either healthspan or the potential age-dependent alteration of specific organs or tissues [3,4]. Moreover, despite the wide range of available genetic manipulations, which are crucial for identifying genes and pathways involved in aging, some of the results obtained by model systems are based on single genetic backgrounds, making general conclusions difficult to draw [5]. In addition, laboratory conditions are generally fixed and do not reflect the fluctuating environment of wild habitats. Finally, although their short generation times and lifespans allow quick data collection, short-lived organisms may exhibit a much greater capacity for lifespan extension compared to humans (up to 10-fold) [6]. However, despite these limitations substantial evidence confirms the power of yeast as a model system, highlighted by the five different Nobel Prizes awarded for yeast-based research (both budding and fission) in recent decades. Part of this success is due to the high conservation of fundamental cellular mechanisms across evolution. Whole-genome sequencing, completed in 1996, 2002 and 2003 for *S. cerevisiae*, *S. pombe* and humans, respectively, confirmed the high degree of homology between yeast and evolutionarily distant species. The availability of whole-genome sequences also enabled genome-wide transcription analysis [7], protein–protein interaction atlases [8], protein localization maps [9], and genome-scale metabolic models [10]. Finally, bioinformatic analysis revealed that *S. cerevisiae* possesses orthologs for approximately 30% of disease-causing human genes [11,12,13,14], suggesting the possibility of using humanized yeasts as models for several human pathologies.

## 2. Laboratory Methods to Investigate Aging in Yeast

### 2.1. Measuring Lifespan

The first demonstration that *Saccharomyces cerevisiae* ages took advantage of the peculiar cell division of this organism. In fact, during the cell cycle, the daughter cell emerges by budding from the mother cell. This peculiarity allows an easy size-based distinction between mother and daughter cells and thus the possibility to count how many times a single cell can divide. Robert K. Mortimer was the first to exploit this asymmetric budding to measure the replicative potential of yeast cells [15]. To do so, the emerging bud was carefully removed by micromanipulation at each cell cycle, and the divisions of its mother cell were counted (Figure 1). This protocol, used to determine the replicative lifespan (RLS), is still in use, albeit with some technological improvements [16]. It has allowed the confirmation or, in some cases, the identification of several genes that play a major role in cellular aging and whose functions are conserved in higher eukaryotes, including mammals. Among these, the roles of the Ras-PKA and TOR-Sch9 pathways are preeminent examples. From a practical point of view, it should be noted that commonly used laboratory yeast strains undergo approximately 20 cellular divisions before entering senescence, with each cell cycle lasting around 2 h. Thus, a single experiment may require up to 40 h of continuous monitoring. To overcome this limitation, researchers usually pause the experiment by placing the Petri dishes at a lower temperature to halt cell division. However, this temperature shift may introduce an experimental bias. In addition, to ensure statistical significance 40 to 50 mother cells must be analyzed in parallel.

Methodological improvements have been introduced to enhance the throughput of this approach. Taking advantage of the scar left on the mother cell, a method allowing enrichment of bud-scar-rich cells has been developed based on the ability of wheat germ agglutinin (WGA) to mark chitin-containing bud scars [17,18]. However, a possible issue of this method is that the continuous growth of progeny cells limits the division capacity of mother cells due to nutrient limitation after a certain number of cell divisions. To overcome these challenges, an alternative is the Mother Enrichment Program (MEP), an inducible genetic system that suppresses the proliferation of daughter cells. The method is based on the expression of the Cre recombinase under the control of the *SCW11* promoter, which is active exclusively in daughter cells being regulated by Ace2p, a transcription factor that selectively localizes to the emerging bud prior to cytokinesis. Upon synthesis, the Cre recombinase excises the essential genes *UBC9* and *CDC20*, which encode for the SUMO-conjugating enzyme and the activator of anaphase-promoting-complex, respectively. The disruption of these genes arrests the division of daughter cells. Cre recombinase is tightly controlled by its fusion to the murine estradiol-binding protein (EBD). In the absence of the hormone, the Cre protein remains localized in the cytoplasm allowing the yeast strain to be easily propagated. However, upon estradiol addition, Cre recombinase translocates to the nucleus, preventing daughter cells from dividing. As a result, only mother cells continue to undergo cellular division, which thereby simplifies their counting [19,20]. In addition, several microfluidic approaches have been developed to monitor mother cell divisions without the need for manual micromanipulation. Although these platforms differ in their specific designs, they all rely on hydrodynamic trapping to retain the larger mother cells, while the smaller daughter cells are continuously flushed away by the media flow (for a comprehensive review of the different microfluidic devices, see [21,22] Table 1 and Figure 1 for a schematic representation of the original method). It has been observed that media used for measuring RLS are typically glucose-rich (such as YPD). Consequently, in these experiments, the cell metabolism primarily relies on glycolysis, a condition that differ from what is observed in most tissues of higher organisms where energy production is mainly based on respiration [23,24]. This idea has been recently challenged by the development of new methods to select aging mother cells [25] and their use in characterizing the metabolism of cells undergoing replicative aging [26]. In fact, the drop in glucose uptake to almost 10% compared to younger cells, the decrease in ethanol production and the increase in oxygen consumption observed suggest the existence of a metabolic shift from a fermentative to a respiratory-based metabolism also during RLS measurement. It must, however, be noted that half of the carbon influx was directed to glycerol and acetate secretion, a condition that is not typically observed during respiration. It is worth noting that acetate has been linked to apoptosis, while glycerol is associated with the stress response [27,28].

A second paradigm for measuring aging, developed by Longo and colleagues, is the chronological lifespan (CLS, [29,30,31]).

Instead of measuring how many times a yeast cell can divide (the replicative potential, RLS), CLS measures how long a non-dividing yeast cell can survive (Figure 1). The standard protocol for measuring CLS starts by streaking yeast cells from a frozen stock onto Petri dishes containing a glucose-rich medium. Once single colonies appear, an isolated colony is inoculated into a synthetic liquid medium with a 1:4 liquid-to-air ratio and incubated at 30 °C with vigorous shaking. Small aliquots are then collected every 1–2 days, serially diluted with sterile water, and plated onto YPD agar plates. The resulting colonies are counted after two days of incubation at 30 °C to determine the number of colony forming units (CFUs). The CFUs at day three is considered 100% (maximum cellular density) and used as a reference for the other time points. During the experiment, the number of CFUs counted at each time point is expressed as a fraction of the CFUs counted at day three (see Figure 1 for a schematic representation).

Several variations have been introduced into the basic protocol to increase both automation and reproducibility. The measurement of the optical density of aging cultures in 96-well plates has thus been proposed as an alternative to the basic CLS protocol [32,33] and used for high-throughput analysis of a library of single-gene deletion strains. The different strains were incubated in 96-well plates containing synthetic medium (SD). At selected time points, a small aliquot was sampled from each culture and transferred into a new 96-well plate containing nutrient-rich YPD medium, where the optical density was determined by absorbance measurement after a fixed incubation period. This approach assumes that the optical density in the YPD culture is directly proportional to the number of viable cells transferred from the aging SD-based culture. The major conceptual difference from the standard method is that, in this case, the 96-well plates are incubated without agitation and with a liquid-to-air ratio far less than the standard 1:4 proportion. Consequently, the respiration-based metabolism observed in the standard CLS protocol may not apply here, implying that the findings obtained by these two different protocols may differ. In addition, the viability of mutants with an altered growth rate can be misinterpreted (see Table 1 for a summary). The solid-plate CLS assay has been developed as a viable alternative to traditional liquid CLS. In this method, cells of the desired strain, which must be auxotrophic for tryptophan, are plated onto multiple synthetic solid medium (SD) lacking this amino acid. At each time point, one plate is supplemented with a tryptophan solution. Surviving cells can then resume growth, allowing the resulting colony-forming units to be scored. Alternatively, a method based on propidium iodide combined with flow cytometry has also been proposed [34] offering some advantages. Subsequent studies have, however, raised concerns about possible bias introduced by this method, thereby limiting its utility [34]. Microarrays coupled with molecular barcode sequencing of pooled strains represent a high-throughput technology that can also be used to assess the viability of non-dividing yeast cells over time. This method has been used to simultaneously screen large collections of yeast deletion mutants [2]. It must be noted that since these experiments are conducted using pooled mutant collections, extracellular factors or metabolites released by one strain could potentially affect the viability of others, causing a non-autonomous cell death/survival.

Aging studies have also been extended to other unicellular fungi. *Schizosaccharomyces pombe* is another very well characterized unicellular eukaryote [35]. Having diverged from the budding yeast hundreds of millions of years ago, the comparison between these two fungi is frequently employed to identify mechanisms conserved throughout evolution. Its use in aging studies is more recent than that of *Saccharomyces cerevisiae* because its symmetrical division makes it difficult to distinguish between the mother cell and daughter cells [36]. This has limited the number of studies focused on replicative lifespan (RLS), although microfluidic-based approaches have recently begun to bridge this gap [37]. Conversely, CLS has been routinely determined in fission yeast, thereby making it possible to compare the results obtained in these two unicellular models of aging [38].

It must also be pointed out that in addition to what each paradigm measures (replicative potential versus survival in the stationary phase), cellular metabolism differs greatly between the two. In fact, while cells during replicative lifespan experiments rely almost exclusively on glycolysis, those in chronological lifespan assays utilize fermentation and respiration [39] as their major energy sources, more closely resembling the metabolic profile of most tissues in higher eukaryotes [40]. It is also worth noting that lowering the glucose availability during RLS measurement, a condition mimicking caloric restriction, whose efficacy in increasing lifespan is well documented in several model systems, extends lifespan only in certain genetic backgrounds. In addition, laboratory strains with different genetic backgrounds display opposite effects in response to caloric restriction upon the loss of mitochondrial DNA [27,41,42]. Interestingly, as described above, the ability to enrich for replicatively aging mother cells has enabled the detailed characterization of the metabolism of replicatively aging cells. The observed drop in glucose uptake, coupled with reduced ethanol synthesis and increased oxygen consumption, suggests that a switch from glycolysis-based to respiratory-based metabolism also occurs during RLS. However, because half of the carbon influx was directed to glycerol and acetate secretion, which differs from what is normally observed during respiration, fundamental metabolic distinctions between CLS and RLS paradigms remain [26,31,43]. Recently, microfluidic-based approaches have been developed to also allow the measurement of RLS in *S. pombe.* Curiously, these findings indicate that replicative lifespan may not represent a valid paradigm for evaluating aging in fission yeast [44], likely due to the absence of asymmetric damage segregation, which is a hallmark of *S. cerevisiae* division. Regarding the CLS assay, yeast cells are grown in synthetic liquid media, where they mitotically divide and reach saturation within 24 to 36 h. After 2 additional days of continuous incubation under vigorous shaking to ensure proper aeration, the cells enter the post-diauxic phase, which is characterized by a high respiratory rate and the massive utilization of non-fermentable carbon sources. Under these conditions, cells remain viable for a duration that depends strictly on both their genetic backgrounds and the specific culture conditions, typically ranging from a few days to a couple of weeks. The duration of viability measures how long yeast cells can survive in the post-mitotic phase. In addition to genetic background, environmental factors such as temperature, pH, acetic acid concentration, and nutrient availability significantly influence chronological survival [31,45,46,47], underscoring the need to validate findings across multiple genetic backgrounds. It must be pointed out that even though yeast liquid cultures in synthetic glucose-rich media reach their maximum cell density within 2–3 days, their metabolism remains high for an additional 4–6 days, with glycogen and ethanol still available when the cells begin to die [48]. These observations confirm that CLS does not simply mimic starvation or a hypometabolic state at least in the first days. It has been argued that the increased concentration of acetic acid observed during CLS and the subsequent decline in extracellular pH may act as an extrinsic aging factor affecting CLS but not RLS [46,49,50]. However, several observations support the physiological role of acetic acid and ethanol in metabolism and aging progression. First, protein oxidation is acetic-acid-dependent and not pH-dependent [51], suggesting a more complex role for acetic acid than simply lowering medium pH. Furthermore, intracellular acidification has been linked to nutrient-dependent signal transduction pathways via Ras signaling and ROS production, confirming the existence of multiple acidification-responsive pathways [52,53,54]. Finally, lactate-dependent medium acidification kills tumor cells maintained in the stationary phase [55], thus suggesting that the role of acidification as a pro-aging cellular driver could be conserved during evolution. A common artifact in classical CLS experiments is the regrowth of aging cells. This happens when a subset of aged cells escapes from quiescence and resumes progression through the cell cycle (GASPing) [56]. While regrowth can easily be detected when the CFUs at a certain time point appears to increase with respect to the previous one, it is more difficult to detect when it occurs early in the experiment. To avoid this artifact, CLS protocols should include repeated water washing of the cultures during the experiments or, alternatively, the use of the solid Trp-plate assay described above. Both these additional methods can eliminate this potential artifact and should therefore be used in parallel [47].

### 2.2. Measuring Stress Resistance and Genome Stability

Given the strong link between cell survival and multiple stress resistance, the latter has been used as a selectable marker to identify genes and mechanisms involved in aging. Heat shock resistance is typically measured by incubating serial dilutions of cells plated on rich medium at 55 °C for a variable time, normally ranging from 30 min to 2 h, and comparing their growth with that of untreated cells.

Oxidative stress resistance assays are performed by resuspending cells in K-phosphate buffer and adding different concentrations of hydrogen peroxide for 30 min. Serially diluted cells are then spotted onto YPD plates and incubated at 30 °C for 2–3 days with the number of colonies reflecting cell survival under stress.

Alternatively, menadione can be used to induce severe oxidative stress through the production of intracellular reactive oxygen species (ROS). Yeast cells counteract this stress by activating antioxidant enzymes, accumulating trehalose, and activating molecular chaperones.

Osmotic stress is an important environmental challenge for yeast in nature, and the ability to adapt to altered availability of water is a crucial feature for living cells. This challenge triggers the High Osmolarity Glycerol (HOG) MAP kinase signaling pathway. In yeast, two different methods are used to activate this stress response: non-ionic osmotic stress, which is usually induced by supplementing the growth medium with 1 M or 1.5 M sorbitol or mannitol, respectively, and ionic/salt osmotic stress, achieved by adding 0.4 or 1 M of NaCl or KCl, respectively [57].

Interestingly, the quality and quantity of nutrients supplied can modulate the efficacy of stressors, suggesting that nutrient availability may regulate cell resistance and longevity [58,59]. Therefore, yeast cultures, which are routinely used to screen collections of mutants, can also be used to evaluate the ability of libraries of substances to modulate resistance to multiple stresses. It is worth noting that these two approaches can also be combined, allowing the identification of nutrient–genotype “synthetic lethality” interactions.

It has long been proposed that genomic instability is one of the drivers of aging, alongside epigenetic alteration, deranged nutrient sensing, mitochondrial dysfunction and telomere attrition [60,61], as well as an enabling factor for several hallmarks of cancer [62]. The role of the somatic mutation rate in aging progression has recently been strengthened by the observation that the mutation rate is inversely proportional to lifespan across several species [63]. The central role of the mutation rate in non-dividing cells has been further supported by the recent availability of single-cell sequencing. Analysis of these data demonstrates that the mutation rate is similar in dividing and non-dividing tissues [64,65]. It is therefore of particular interest that different types of mutations can be easily identified at very low cost using the yeast model. To this end, the above-described chronological lifespan measurement can be combined with the detection of different types of DNA mutations [66], ranging from point mutations to large chromosomal rearrangements. Forward mutation rates can be measured in different ways, the most common of which are resistance to 5-fluoroorotic acid (5-FOA), canavanine and alfa-factor. 5-FOA is a non-toxic substance that can be converted into the highly toxic 5-fluorouracil by the uracil biosynthetic pathway. The central gene involved in this process is *URA3*; therefore, this method basically measures any mutation causing a loss of function of the orotidine-5′-phosphate decarboxylase protein, the enzyme coded by the *URA3* gene [67]. The second method to assess the mutation rate in yeast is based on Can1p permease. Wild-type *CAN1* codes for the membrane-bound arginine permease that mediates the cellular uptake of both arginine and canavanine. Since canavanine is toxic at a concentration of 60 mgL^−1^, cells incubated in canavanine-containing media will not grow unless a de novo mutation inactivates the permease. Although several types of mutations can occur at the *CAN1* locus, sequencing has demonstrated that this method mainly measures point mutations in haploid cells (65%), whereas frameshifts and more complex mutations are less common (35%) [40]. Alfa-factor blocks the cell division of *MATa* cells and prepares them for mating. Any mutation affecting the signaling cascade that responds to this factor is therefore capable of restoring cell division. The target genes include *STE2*, which codes for the alfa-factor G-protein-coupled receptor, and several elements of the downstream MAPK signaling cascade. However, this method cannot be used for wild-type strains because of their mating-type switch ability [67,68,69]. To specifically identify point mutations, the reversion of amber or ochre stop codons can be measured. A simple method uses the standard laboratory yeast strain DBY746, which carries a C to T transition at nucleotide position 403 of the *TRP1* coding sequence. This transition introduces a premature stop (amber) codon within the ORF, resulting in tryptophan auxotrophy. The experimental protocol resembles that for acquired canavanine resistance. However, since only a mutation reverting the *trp1-289* allele (a reverse mutation) will rescue the phenotype, a larger number of cells must be used (10^8^), these are washed and plated on selective medium (SDC-Trp) to detect enough revertants [40].

To score frameshift mutations, a specifically engineered yeast strain is required. The *LYS2* locus of this tester strain was digested with *Bgl*II, filled-in, and ligated to generate an insertion, consequently causing a frameshift within the coding region of the gene. The resulting lysine auxotrophy can then be rescued only by a de novo insertion or deletion mutation capable of restoring the wild-type ORF.

Gross chromosomal rearrangements (GCRs) also require an engineered yeast strain. In this system, the *HXT13* gene is replaced with the *URA3* marker. Because fluoroorotic acid is toxic to cells expressing *URA3* and canavanine is toxic to cells expressing wild-type *CAN1*, only the cells that have lost the approximately 7.5 kb DNA fragment comprising *URA3* (at the *HTX13* locus) and *CAN1* can survive in this double-selective medium [40]. Subsequent sequencing of the surviving cells confirmed the deletion of this chromosomal region (see Table 2 for a summary). An alternative, unbiased approach to these single gene analyses would be to compare the genome of parents and offspring by whole-genome or exome sequencing [70]. However, although the cost of next-generation sequencing is continuously decreasing, its routine use for screening mutant libraries or small molecules remains impractical.

## 3. Aging Genes and Pathways

### 3.1. RAS-PKA Pathway

Interest in this pathway among researchers in the field of aging started with the observation that the expression of retroviral v-Ha-RAS in yeast resulted in RLS extension [71], an observation that is consistent with its ability to immortalize mammalian fibroblasts [72]. Later, RLS studies curiously demonstrated that the deletion of *RAS2* decreased RLS, while the deletion of *RAS1* had the opposite effect [73], and deletion of *ras1* in *S. pombe* does not increase the lifespan under nutrient limitation [74]. However, the deletion of *RAS2* doubles the chronological lifespan (CLS) of yeast cells and significantly increases their resistance to multiple stresses [75]. Ras (Rat sarcoma) proteins are part of the monomeric G-protein superfamily, a group of proteins characterized by the presence of the G domain—an evolutionarily conserved amino acid sequence capable of binding and hydrolyzing the nucleotide GTP. Monomeric G proteins, of which Ras proteins are no exception, cycle between an inactive, GDP-bound state and an active, GTP-bound state, thereby regulating yeast cell metabolism and proliferation [76,77,78]. In *S. cerevisiae*, increased Ras activity stimulates adenylate cyclase to produce cAMP, which in turn activates protein kinase A (PKA). PKA phosphorylates specific serine and threonine residues on target proteins in response to these elevated secondary messenger levels [78,79]. The role of the Ras-PKA pathway in aging was first identified in yeast, where the presence of a *ras2* null allele results, as mentioned above, in a twofold extension of the chronological lifespan (CLS) and a concurrent increase in resistance to multiple stresses [75,76]. The role of the PKA pathway was more recently confirmed in *S. pombe*. Specifically, deletion strains of git3 (a glucose G-protein-coupled receptor), git5 (a G-protein β subunit), and pka1 (the PKA catalytic subunit) exhibit an extended CLS in this organism [80]. In *S. cerevisiae*, Ras2p activation primarily triggers two signaling branches: the protein kinase A (PKA) and the mitogen-activated protein kinase (MAPK) pathways. Accumulating evidence identifies the PKA axis as the principal regulator of longevity, as demonstrated by the extended lifespan of yeast strains carrying a transposon integrated *mTn::cyr1* allele [81]. The *S. cerevisiae* PKA regulatory subunit is coded by a single gene, *BCY1*, the manipulation of which affects longevity and stress resistance. Conversely, three distinct genes—*TPK1*, *TPK2*, and *TPK3*—code for the catalytic subunits, which display both overlapping and specific substrate preferences [82]. The pro-aging effects of yeast PKA are largely mediated through the inhibition of Msn2p and Msn4p, two pivotal stress-responsive transcription factors. Indeed, the deletion of *MSN2* and *MSN4* effectively abrogates the longevity benefits conferred by Ras2p deficiency [83]. Furthermore, loss-of-function mutations in adenylate cyclase—the upstream activator of PKA—similarly augment yeast survival, corroborating the PKA pathway’s status as a central, Ras-dependent aging regulator. Mechanistically, PKA activation leads to the suppression of the Rim15p kinase. The inhibition of Rim15p, in turn, prevents the activation of the Gis1p, Msn2p, and Msn4p transcription factors. Under conditions of low PKA activity, these factors bind to post-diauxic shift (PDS) elements and stress response elements (STREs), thereby driving the expression of cytoprotective genes. These include genes coding for cytosolic catalase, superoxide dismutases (*SOD1* and *SOD2*), heat shock proteins (*HSP*s), and the multi stress response gene *DDR2* [84,85,86,,87]. Inhibition of the PKA pathway in *S. pombe* activates the Sty1 pathway, and Sty1 overexpression extends the CLS in this organism. Sty1, which is the terminal component of a mitogen-activated protein kinase (MAPK) pathway, is a stress-activated protein kinase regulating cellular survival. Once activated by phosphorylation, Sty1 accumulates in the nucleus and activates several transcription factors that coordinate the cellular response to multiple stresses [88,89,90,91]. Although no biochemical correlation exists, these observations suggest a functional similarity between the *S. cerevisiae* serine/threonine kinase Rim15p and the MAP kinase Sty1 in *S. pombe*. Many studies have confirmed the observations made in yeasts in higher eukaryotes [92,93,94]. In fact, the deletion of the catalytic PKA subunits confers health benefits in mice [92,93,94]. Similarly, deletion of adenylate cyclase 5 ameliorates cardiac aging and increases the lifespan of mice [95].

### 3.2. TOR-SCH9 Pathway

Rapamycin, initially purified from the soil bacterium *Streptomyces hygroscopicus* for its antifungal activity [96], was eventually characterized using yeast genetics. It forms a complex with Fkbp12p, a protein-folding chaperone conserved from yeast to humans, which in turn inhibits a protein kinase subsequently named Target of Rapamycin (TOR).

Interestingly, the systematic isolation of long-lived yeast mutants via a large-scale collection of single-gene deletion strains and RLS assays identified the TOR axis as a potent pro-aging pathway—a finding later confirmed by CLS assays. These RLS experiments also validated the pro-aging function of Sch9p, which had been previously identified by CLS assays [97,98]. Furthermore, it has been demonstrated that decreased TOR activity increases respiration and mitochondrial protein expression, activating an adaptive response capable of significantly increasing survival [32,99] (Figure 2).

Molecular and biochemical studies have revealed that the Tor protein serves as the catalytic subunit of two distinct complexes: TORC1 and TORC2. These complexes possess distinct functions, but only TORC1 is sensitive to rapamycin [100]. In yeast, two different genes, *TOR1* and *TOR2*, code for the catalytic subunits of TORC1, while TORC2 contains only Tor2p [100]. They share 67% identity, but only *TOR2* is essential for viability. TORC1 is a multimeric complex composed of one of the two serine/threonine protein kinase, Tor1p or Tor2p, in combination with three regulatory subunits, Lst8p, Tco89p and Kog1p [100]. Given its role as a regulator of longevity and its conservation from yeast to humans, the molecular network of the TOR pathway has been characterized in detail. Nonetheless, this molecular characterization has also revealed some divergent characteristics throughout evolution. For example, while rapamycin treatment inhibits the respiration of mammalian cells, it has the opposite effect on yeast cells [100,101,102].

TORC1 is activated by both Rag GTPase-dependent and Rag GTPase-independent mechanisms [103,104,105]. The former pathway involves Gtr1p and Gtr2p, the yeast Rag GTPase orthologs, which form an active heterodimer linked to the vacuolar membrane. This localization is mediated by the EGO complex (EGOC), which acts as a binding platform for Gtr1p/Gtr2p; once activated, this heterodimer binds Kog1p and stimulates a transient increase in TORC1 activity [106,107]. Gtr1p is regulated by the guanine nucleotide exchange factor Vam6p and the GTPase-activating protein (GAP) SEACIT, while the Lst4p-Lst7p complex functions as a GAP for Gtr2p [107]. Concurrently, TORC1 can phosphorylate Lst4p, establishing a negative feedback mechanism that prevents TORC1 hyperactivation [108].

The Rag GTPase-independent regulation of TORC1 involves the phosphatidylinositol 3-phosphate-binding protein Pib2p and the Vps15p-Vps34p complex [109]. Both mechanisms are activated by amino acid availability, which further regulates TORC1 through pathways involving the Rab GTPase Ypt1p and the protein kinase Gcn2p [110]. From a metabolic perspective, when activated by nutrients, particularly amino acids, TORC1 promotes anabolic processes and cell growth while inhibiting catabolic processes and stress responses. Indeed, TORC1 regulates protein synthesis, sphingolipid metabolism, cell cycle progression, the stress response, and autophagy [102,103,104,105].

In the presence of various stresses, such as heat shock and nitrogen limitation, TORC1 regulates the activity of protein phosphatase 2A (PP2A) and PP2A-like phosphatase complexes via Tap42p [111,112,113]. Specifically, active TORC1 phosphorylates Tap42p, which directly modulates the activity and localization of these phosphatase complexes. Upon nitrogen starvation, TORC1 is inactivated and cannot interact with the Tap42-containing complexes; consequently, the Tap42p-PP2A complex is released into the cytosol where the phosphatase becomes active, whereas subsequent Tap42 dephosphorylation releases the complex, terminating the Tap42-dependent phosphatase activity [111,114,115,116,117].

Similar phenomena have been described in *S. pombe* [118]. The fission yeast also possesses two distinct TOR complexes: TORC1 and TORC2. While TORC2 is dispensable for cell proliferation, it is required for sexual differentiation, actin function and the stress response [119]. Conversely, TORC1 activates anabolic processes, such as protein synthesis, and inhibits catabolic processes, including autophagy [120]. Interestingly, the ATP analog Torin1 blocks both TORC1 and TORC2 complexes in *S. pombe*, thereby prolonging CLS [119,121]. Because Torin1 treatment blocks cell proliferation, mutants capable of overriding this block were isolated [119]. Subsequent characterization of these mutants identified components of the endosomal sorting complexes required for transport (ESCRT), some of which are involved in autophagy [121]. Notably, components of the Golgi-to-vacuole molecular trafficking machinery are also required for the TORC1-dependent regulation of the GATA transcription factor in budding yeast [122,123].

One of the major TORC1 effectors involved in yeast aging is the AGC protein kinase Sch9p, which links nutrient availability with cell cycle progression, cell size and autophagy. Furthermore, Sch9p modulates stress resistance, sphingolipid metabolism and protein synthesis [124,125,126,127].

The *SCH9* gene (named for Scott Cameron *Hind*III library clone number 9 [127]) was initially identified as a multicopy suppressor of the growth defect caused by a temperature-sensitive *cdc25* allele [128]. Sch9p shares structural and functional characteristics with the mammalian AKT1 and S6K1 protein kinases. Sch9p co-localizes with TORC1 on vacuolar membranes [129]; this membrane localization is mediated by its interaction with Ptdins(3,5)P2 [130]. However, under carbon source limitation, Sch9p disperses from these sites into the cytosol [131].

Sch9p is activated through the phosphorylation of multiple amino acid residues by various protein kinases. These include the paralogous kinases Pkh1p, Pkh2p and Pkh3p, which are orthologs of the mammalian Phosphoinositide-Dependent protein Kinase 1 (PDK1) [132] and phosphorylate Sch9p at Thr570. PDK1 acts as the main downstream effector of phosphoinositide 3-kinase (PI3K), whereas the yeast orthologs Pkh1-3 are activated by sphingolipids [132,133]. Additionally, TORC1 phosphorylates multiple residues within the hydrophobic motif (HM) of the Sch9p C-terminal region [134], while the cyclin-dependent protein kinases (CDKs) Bur1p and Pho85p phosphorylate Thr723 and Ser726 [135,136]. Pho85p activity is, in turn, regulated by phosphate levels [137,138,139]. *S. pombe* possesses two orthologs of *SCH9*, namely, *sck1* and *sck2*, which encode AGC kinase family members whose phosphorylation by the TORC1 complex has been demonstrated [140]. Interestingly, the deletion of *sck2* extends the chronological lifespan only in the presence of wild-type Sty1 MAP kinase [90,140,141], thereby confirming the functional crosstalk between the PKA, MAPK and TOR signaling pathways in fission yeast.

Consequently, Sch9p plays a critical role in nutrient response mechanisms by integrating signals from at least two different pathways: the TORC1-S6K axis and the sphingolipid-dependent Pkh1/2/3 pathway. This integration allows Sch9p to adapt its phosphorylation status and activity accordingly. A consequence of this dual regulation is the alteration of Rim15p subcellular localization under partial depletion of specific amino acids (such as threonine, serine and valine), a condition known to extend yeast lifespan [58].

Conversely, Sch9p activity can also be inhibited through the Snf1p-mediated phosphorylation of Ser288 [142,143].

Sch9p directly or indirectly regulates numerous downstream effectors. These include protein kinase A (PKA), where Sch9p prevents the phosphorylation and consequent activation of its regulatory subunit Bcy1p [144], as well as various other protein kinases and transcription factors [144]. Among these effectors, Rim15p plays a central role in the stress response and aging. The inhibition of the Sch9p kinase leads to the translocation of Rim15p into the nucleus, where it activates transcription factors such as Msn2/4p and Hsf1p. This activates the expression of genes containing stress-responsive elements (STREs), heat shock elements (HSEs), or post-diauxic shift (PDEs) elements in their promoter, such as *HSP26*, *HSP12* and *SOD2* [6]. Consistent with these findings, deletion of *SCH9* significantly decreases the overall mutation frequency and DNA damage during chronological aging [145]. Sch9*Δ* cells show enhanced *SOD2* expression and higher oxidative stress resistance, leading to decreased superoxide-induced DNA damage. Moreover, the error-prone DNA polymerase subunit Rev1p is less active in the *sch9Δ* mutant due to a Gis1p-dependent reduction in *REV1* expression [146]. Interestingly, *tor1Δ* or *sch9Δ* cells exhibit an elevated respiratory capacity that transiently increases ROS levels, thereby promoting longevity through a mechanism reminiscent of mitochondrial mitohormesis [146,147].

Furthermore, Sch9p influences the proteasomal and autophagic degradative systems through the regulation of proteasome assembly [148,149].

Consequently, the loss of Sch9p function extends the yeast lifespan [6,31,33]. Interestingly, the protracted G1 phase characteristic of *sch9Δ* mutants is rescued by PKA hyperactivation, suggesting a functional redundancy between these two pathways. In addition, the simultaneous downregulation of these pathways, often triggered by nutrient deprivation, results in a robust upregulation of stress defense genes [30,83].

The central role of TOR as a pro-aging pathway that regulates anabolic processes, first identified in yeast, has also been extensively confirmed in higher eukaryotes. In higher organisms, the mTOR axis integrates various upstream inputs, including the insulin/IGF-1 signaling cascade, and remains highly sensitive to intracellular amino acid concentrations, a feature originally discovered in mammals and confirmed in yeast [58,150,151,152,153]. Consequently, pharmacological or genetic attenuation of mTOR signaling consistently enhances longevity across diverse phylogenetic taxa [32,154,155,156,157,158,159,160,161,162]. In *Caenorhabditis elegans*, null mutations in *let-363* (the mTOR ortholog) or its knockdown by RNA interference (RNAi) result in a significant lifespan extension [154]. Similarly, in *Drosophila melanogaster*, the tuberous sclerosis complex (Tsc1/2) acts as a negative regulator of TOR [157]. Consequently, the overexpression of *dTsc1* or *dTsc2*, as well as the expression of dominant-negative forms of *dTOR* or *ds6k*, promotes longevity [158].

Inhibition of mTOR signaling extends both the median and maximal lifespan in mice [160,161,162]. Furthermore, rapamycin administration in mice and rats preserves the ovarian reserve and extends the reproductive lifespan. This effect is mediated through the suppression of mTOR signaling and the concomitant upregulation of sirtuins, specifically SIRT1 and SIRT6 [150,155,159]. Similarly, inhibition of S6 kinase extends both healthspan and lifespan in mice [163], whereas its deletion regulates inflammaging [155,163].

## 4. Nutrients and Longevity

As described, the inhibition of both the Ras-PKA and TOR-sch9 pathways (either alone or in combination) promotes longevity. This effect is mainly achieved by enhancing stress resistance through the activation of the transcription factors Hsf1p, Msn2p and Msn4p [164,165] or the translocation of Msn2p and Msn4p into the nucleus. For example, *SOD2* expression doubles in *ras2Δ* strains, leading to increased survival [58,166]. Emerging evidence suggests that lifespan extension is promoted by the ability to resist multiple stressors, in particular oxidative stress. However, it must be noted that the exogenous overexpression of superoxide dismutases and catalases fails to replicate the substantial longevity gains achieved through the deletion of *RAS2* or *SCH9* [83,,167]. This suggests that while the mitigation of oxidative stress is a vital component of the longevity response, the regulatory network of aging involves a broader, more complex set of physiological recalibrations to a dynamically changing environment. A striking example is the impact of caloric restriction and nutrient deprivation on longevity.

Nutrient availability is the primary exogenous regulator of lifespan in simple eukaryotes, and its relevance is strengthened by the observation that caloric restriction is an effective method to increase lifespan in higher eukaryotes [168,169,170]. In *S. cerevisiae*, the molecular response to the chemical environment is mediated by specialized signal transduction pathways that respond to specific nutrients: the presence of fermentable carbon sources triggers the Ras–adenylate cyclase (AC)–PKA pathway, whereas nitrogenous compounds regulate the Pkh/Pdk and Tor-Sch9/S6k signaling branches [58]. Pharmacological or genetic downregulation of these nutrient-sensing nodes is strongly associated with the extension of both lifespan and stress resistance. These longevity-promoting effects are mechanistically linked to several downstream cellular shifts, including the activation of the stress response transcription factors Msn2p and Msn4p, enhanced expression of endogenous antioxidant enzymes, reduced accumulation of DNA lesions, and a prolonged lifespan [58,171]. Recent investigations have sought to define the specific molecular mechanism of caloric restriction (CR), which is typically achieved by lowering glucose availability (e.g., reduction to 0.5%) and of limiting nitrogenous sources. The results show that nitrogen restriction fosters longevity by upregulating autophagy, RNA processing, and mitochondrial bioenergetics [172,173,174,175,176,177,178]. While the restriction of different carbon sources (e.g., glycerol vs. glucose) may yield divergent outcomes [6,179], the fundamental longevity-associated response involves the coordinated regulation of glycolysis, the tricarboxylic acid (TCA) cycle, and oxidative phosphorylation [58]. It is well known that the increased longevity and stress resistance observed with caloric restriction in yeast can be linked to the modulation of the Ras/cAMP/PKA/Rim15/Msn2/4 and Tor/Sch9/Rim15/Gis1 pathways [31]. However, it must be noted that cells with impairment in both pathways are still sensitive to caloric restriction, suggesting that a more complex protein network is affected by this dietary intervention. One of the possible additional mechanisms is the modulation of autophagy. It is known that caloric restriction inhibits TORC1, which subsequently leads to increased autophagy [175,176,180]. More than 40 autophagy-related genes have been identified in yeast, most of which are conserved in higher eukaryotes [180]. Several conditions, including caloric restriction, can modulate autophagy through phosphorylation of its components [181]. In addition, nutrient deprivation can impinge on histone acetylation via mechanisms involving Sin3p and Rpd3p [182,183]. However, an autophagy-independent mechanism has also been identified in certain mutants. The authors suggest that, in these strains, the effect of caloric restriction depends on redox homeostasis and metabolic adaptation rather than on autophagy regulation [184].

The degradation of aberrant proteins through protein quality control, ubiquitination and protein turnover has also been proposed as a possible mechanism affected by caloric restriction [185].

Since caloric restriction is normally achieved in yeast by reducing the glucose concentration in the medium, the role of sugar availability has been studied in greater detail. As expected, glucose and fructose overload results in shorter CLS. It was also observed that the deletion of *TOR1* or of *VAC8* (coding a vacuole-associated protein) improved survival and altered the intracellular ceramide concentration [186]. On the contrary, ceramide accumulation by drug treatment decreased lifespan, while the pharmacological inhibition of sphingolipid synthesis had the opposite effect [186]. In addition, a specific lipidome was detected in caloric-restricted cells [187]. These results suggest a link between sugar availability, lipid metabolism and CLS, potentially mediated by the intersection of sugar toxicity and lipid/ceramide production.

Since it is known that the inhibition of TORC1 reduces the rate of protein synthesis, thereby increasing lifespan, it is not surprising that reducing amino acid availability may extend CLS [110,171]. In addition, mutations that impair ribosome biogenesis exert similar effects, and the reduced synthesis by RNApol III, which is responsible of tRNA transcription, yields the same outcome [188,189,190].

Yeast genetic manipulation relies heavily on auxotrophic markers, which are mutated genes that prevent the yeast from synthesizing an essential nutrient, typically an amino acid. These amino acids are thus essential for the growth of laboratory yeast. Alternatively, the use of dominant heterologous markers such as *KanMX*, *HphMX or NatMX* eliminates the need for auxotrophies. In addition, marker-less genome engineering can be achieved using the CRISPR/Cas9 methodology [191].

Nonetheless, the effect of caloric restriction was evaluated in combination with the availability of essential amino acids in auxotroph yeast strains. The authors observed a longer lifespan with glucose restriction than with essential amino acid restriction. In addition, higher hydrogen peroxide sensitivity and an increased rate of mutagenesis were observed when essential amino acids were depleted [192]. Another study compared a panel of amino acid auxotrophs. Interestingly, varying effects were observed under auxothrophic starvation [193]. However, the distinct effects of amino acid auxotrophies on longevity and stress resistance strengthen the idea that amino acids are not merely protein building blocks or substrates to build other molecules. This consideration is supported by the evidence that arginine and leucine act as TOR activators, whereas valine, serine and threonine regulate the phosphorylation state of Sch9p via the modulation of Pkhs and TOR [58].

Many other genes and substances have been found to extend CLS under specific conditions; however, although interesting, most of them are still awaiting confirmation in higher organisms (for a review, see [2]).

## 5. Determinants of Longevity in Humans

Establishing a definitive correlation between specific human allelic variants and the long-lived phenotype has proven to be exceptionally challenging and, so far, limited to a few loci. Furthermore, while a significant body of literature posits that senescence possesses a fundamental genetic component, empirical evidence regarding the heritability of longevity remains somewhat elusive. However, the assumption that genetics accounts for 20–30% or even less of human longevity has recently been challenged. In fact, after removing confounding of extrinsic factors, such as accidents, the estimated heritability of longevity increases up to 50%, a percentage comparable to other complex human traits and to what has been observed in other species [194].

On the other hand, several studies confirm the central role of diet in both animal models and humans. Caloric restriction (CR), the most widely known dietary intervention, can increase lifespan in mammal models and, potentially, in humans. CR inhibits anabolic hormones and inflammatory cytokines, reduces cell proliferation and oxidative stress, and promotes both DNA repair mechanisms and autophagy [195,196]. Since many model systems used for aging studies display a positive correlation between caloric restriction and longevity, investigating the molecular effects of CR in these systems is highly valuable for gaining insights into human aging. Studies in yeast have shown that the deletion of *SCH9*, the yeast ortholog of S6/AKT, delays the characteristically observed age-dependent increase in the mutation rate, suggesting a role for the TORC1 complex in this process [66,145]. Interestingly, long-term rapamycin treatment reduced the mitochondrial mutation frequency in aging mice [197] and decreased cancer incidence and progression [198] while increasing autophagy, thus suggesting the existence of similar mechanisms in mammals. Recently, the effect of rapamycin on the immune system was also assessed, revealing a significant reduction in γδ T cells producing the pro-inflammatory cytokine IL17 [199]. However, translating these observations to humans remains challenging due to the lack of validated endpoints [200]. In addition, chronic rapamycin treatment triggers glucose intolerance and dyslipidemia [201], while its role on testicular atrophy remains more controversial, thereby limiting the use of rapamycin as an anti-aging drug [202,203]. In addition, the immunosuppressive effect of rapamycin can increase the risk of infections and affect wound healing in the general population [204].

Beyond rapamycin treatment, the role of TOR in human longevity has been supported by a recent case–control study in which a German longevity cohort was screened for rare genetic variants and compared to a large set of younger controls. Notably, several of the rare mutations identified in the longevity group clustered in the mTOR signaling pathway [205].

The role of the Ras and PKA pathways has also been confirmed in mammals.

In fact, the insulin/IGF-1 signaling cascade, which is one of the upstream activators of Ras, is arguably the best-known pathway regulating mammalian longevity [206]. IGF1-R activation inhibits the forkhead box O3 (FOXO3) transcription factor, and the *FOXO3* genotype has been strongly associated with human longevity [207], as confirmed by association studies [208,209,210]. Importantly, both the FOXO3 and IGF1-R pathways modulate survival and stress resistance in response to dietary restriction [211,212].

Given the strict relationship between FOXO3 and insulin/insulin-like growth factor 1 signaling [213,214], it is interesting to note that increased expression of *FOXO3*, which can be obtained by enhancing ketone body production or administration, or by inhibiting the IGF-1 signaling cascade, increases stress resistance primarily by activating the transcription of antioxidant genes, such as *SOD2* and *CAT* (catalase) [215,216]. It must be noted that the downregulation of the Ras/cAMP/PKA or Tor/Sch9 pathways, which is required to extend yeast lifespan, provokes the upregulation of the stress-resistance transcription factors Msn2/4p and Gis1p as well as of the serine/threonine kinase Rim15p. These factors collectively regulate several stress-resistance genes including the mitochondrial superoxide dismutase gene *SOD2* [31].

PKA is expressed ubiquitously in mammalian tissues and is activated by multiple mechanisms; it plays a central role in linking signal transduction downstream of G-protein-coupled receptors with the regulation of metabolism and triglyceride storage (Figure 3). As in lower eukaryotes, it is a heterotetramer composed of two regulatory and two catalytic subunits. Since multiple catalytic and regulatory subunits exist, the exact composition of the holoenzyme can vary according to the tissue-specific expression of these different isoforms. Interestingly, RI isoforms lack a phosphorylation site, which may affect their avidity for the catalytic subunits [217].

**Figure 3:**
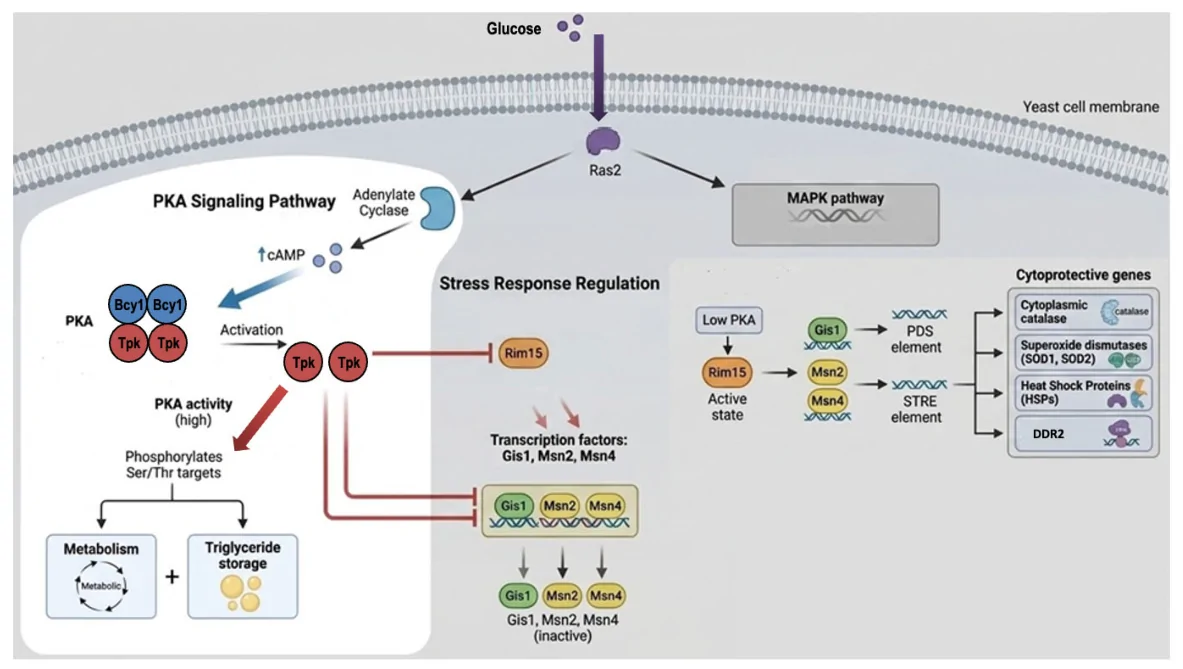
Yeast Ras-PKA signal transduction pathway. Ras2 protein transduces extracellular nutritional stimuli, primarily glucose abundance, into intracellular metabolic and physiological responses. The activation of Ras2 leads to the production of cyclic AMP by adenylyl cyclase; cAMP then binds to the regulatory subunits (Bcy1) of protein kinase A (PKA), triggering their dissociation and thereby enabling the activation of the kinase itself. Once activated, PKA phosphorylates several downstream targets and, in particular, inhibits Rim15, which would otherwise positively regulate the transcription factors Gis1, Msn2, and Msn4. Upon binding to PDS (Post-Diauxic Shift) and STRE (STress-Responsive Element) sequences, these transcription factors activate the expression of genes involved in cellular survival and stress response such as certain heat shock proteins (HSPs), cytosolic catalase, multi stress resistance (DDR2) and both superoxide dismutases (SOD1, SOD2).

Although there is still no direct evidence linking PKA to human aging, the loss of function of the RIIβ regulatory subunit in mice results in increased insulin sensitivity, a lean phenotype and increased longevity [93]. In addition, mice lacking the Cβ isoform of the catalytic subunits are protected from weight gain, enlarged livers, and cardiac dysfunction [218], while enhanced hematopoietic stem-cell-based regeneration is also observed upon stress [219].

Other human studies [220] found a significant association with longevity and the *TOMM40/APOE/APOC1* gene cluster (19q13.11–19q13.32) and confirmed the *FOXO3* gene (6q21). Apolipoprotein E (*APOE*) has two specific allelic variants known to influence longevity through their association with disease: ε2 and ε4 [221]. The ε2 allele promotes longevity largely by decreasing the risk of cardiovascular and Alzheimer diseases, whereas the ε4 variant exerts the opposite effect [222]. Consistently, the ε2 allele is enriched in centenarians, whereas the ε4 allele is significantly diminished [223,224,225].

Bin Jumah et al. identified more than 50 human genes associated with longevity [224,226]. Interestingly, half of these genes are involved in the oxidative stress response, lipid metabolism and the DNA damage response. Notably, oxidative stress is responsible for driving both genomic instability and lipid peroxidation.

Studies on centenarians have identified enhanced DNA repair activity, a lower frequency of genomic damage, and longer telomeres within this population [227], suggesting that DNA repair efficiency and telomerase expression play a pivotal role in longevity.

Furthermore, stem cell exhaustion and immune senescence have also been proposed as major drivers of aging in mammals and humans [219].

In addition, nutrients and nutrient-dependent pathways may contribute to low-grade, systemic chronic inflammation, a condition that can also be triggered by xenobiotics, dysbiosis, obesity, physical inactivity, chronic infections, poor diet and sleep disturbances. This sustained inflammatory state, which underlines the process of inflammaging, is more pronounced in short-lived individuals and has been associated with advanced age and susceptibility to disease [220].

From this perspective, it appears that while basic cellular mechanisms have been conserved during evolution from yeast to mammals, other mechanisms are specific to more complex species [225,226,228,229]. Stress resistance, genome stability, diet and its composition, caloric restriction, nutrient signaling pathways, and mitochondrial disfunction are examples of common ground between yeast and humans. Consequently, these shared features reinforce the need to deeply explore the fundamental molecular and cellular determinants of longevity using this simple model organism. Of course, other aspects of longevity, such as low-grade inflammation, immune-senescence, inflammaging, stem cell exhaustion, and specific components of lipid metabolism, require the use of more closely related mammalian model systems [229,230,231].

## 6. Conclusions

The aging process is a highly intricate and multifactorial phenomenon with huge economic and social implications. Most of the recent literature suggests that important determinants of life extension may not reside in the genomic sequence itself, as suggested by the several discordant phenotypes observed in monozygotic twins, but rather by the dynamic modulation of genomes and gene products. This regulation is driven by a complex interplay of several exogenous factors, most notably nutrients and signaling molecules. This paradigm shift from static genetic determinism to adaptive signaling presents a strategic advantage for developing interventions aimed at extending survival and enhancing healthspan, because the genome remains largely immutable, while metabolic pathways are highly plastic, and targeted modulation of the biochemical signals that govern their activation is possible. Therefore, a detailed understanding of the interplay between genomic determinants, environmental inputs, and lifestyle is a key point of healthy aging research field. In this scenario, the in-depth investigation of the molecular mechanisms may take advantage of simple model systems, which offer greater genetic tractability and faster generation times. Yeast, the simplest eukaryotic model system, is an easy to manage and at the same time a powerful model of aging. Limited ethical constraints, cost-effectiveness, and the extensive array of available bioinformatic, genetic, and molecular tools are some of its benefits. Furthermore, the short lifespan enables the longitudinal monitoring of multiple generations within a practical timeframe. The yeast model not only possesses these advantages but also offers the unique availability of billions of isogenic individuals. This characteristic facilitates a precise distinction between environmental and genetic determinants of observed phenotypes. Following the landmark discovery of Ras as a fundamental pro-aging pathway, yeast has served as a primary model for identifying critical longevity-related genes and signaling cascades, most notably the Sch9-TOR axis—the second evolutionary-conserved pro-aging pathway. The regulatory dependence of these pathways on nutrient sensing led to the characterization of specific roles played by individual nutrients, such as glucose and specific amino acids. Significantly, the discoveries initially made in yeast regarding these nutritional influences have subsequently been validated in mammalian models. Comparative analysis reveals that some identified mechanisms involved in longevity are present in a wide range of species, confirming the usefulness of this system. Although some critical pathways have already been discovered, there are still unanswered questions: (1) Is the damage caused by oxidative stress at least partially reversible? (2) Are there alternative cellular pathways that can activate stress resistance despite the presence of pro-aging signals? (3) Which environmental conditions or substances (nutrients or drugs) can activate them? Answering these questions could suggest interventions not only to delay aging but also to promote rejuvenation. We believe that to answer these still open questions, we will need, once again, the help of yeast.

## Figures and Tables

**Figure 1:**
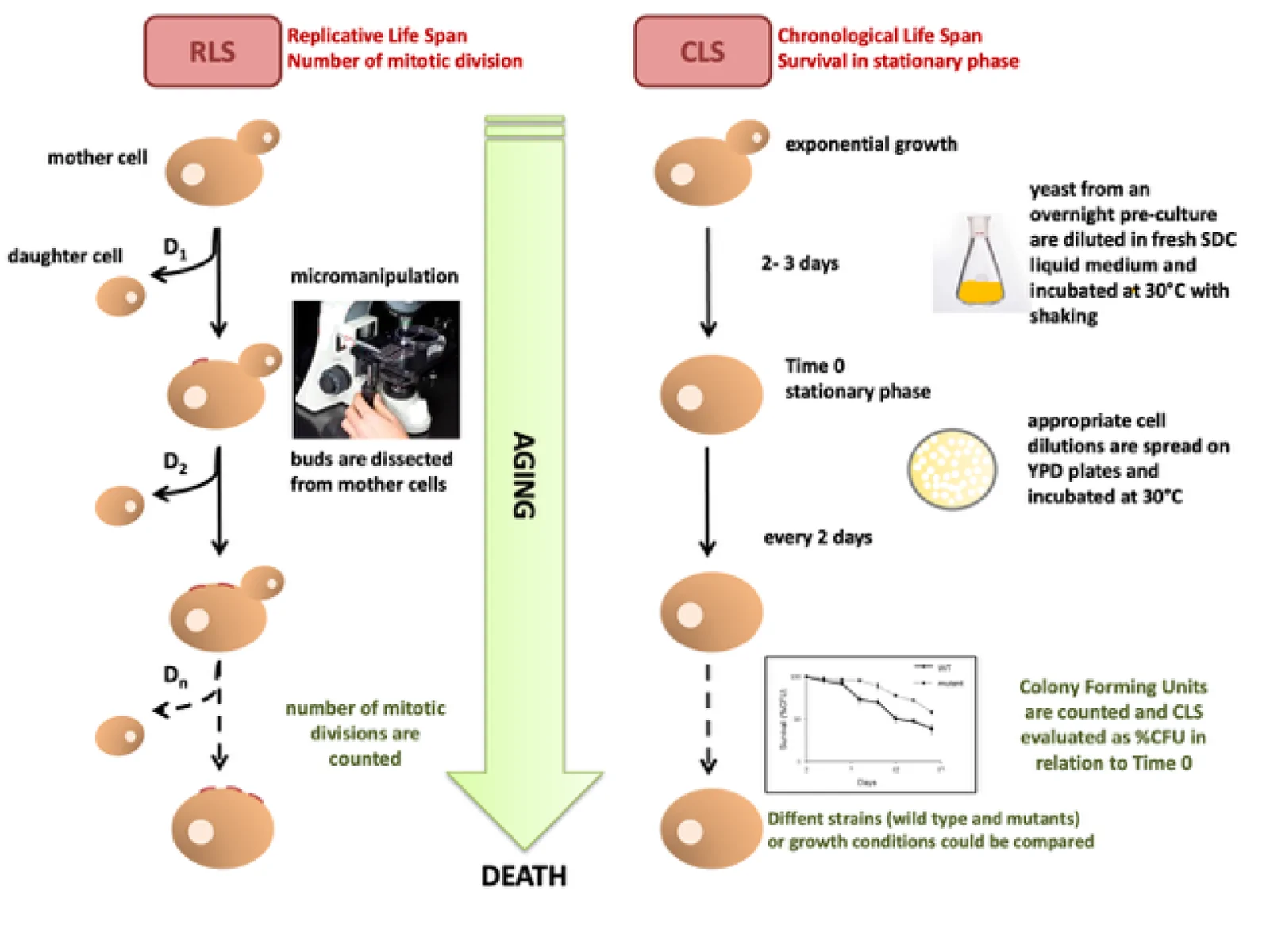
Schematic representation and comparison of the original protocols used for RLS and CLS.

**Figure 2:**
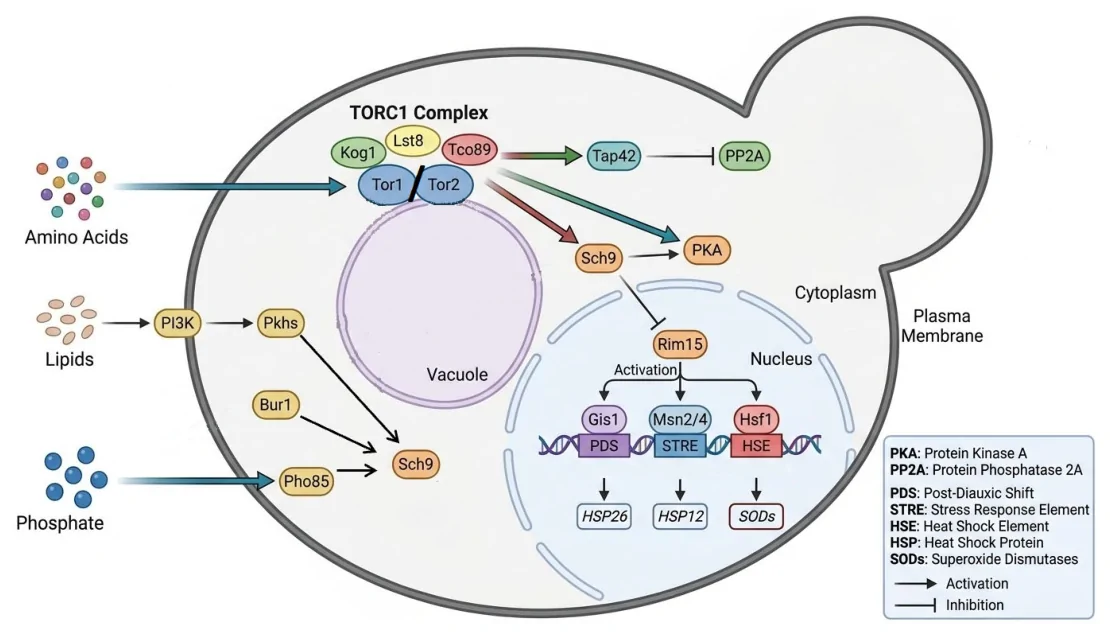
Amino acid, lipid, and phosphate availability triggers distinct kinase signaling pathways that converge upon Sch9 to modulate the Rim15-mediated stress response. The Sch9 pathway is a key longevity pathway in yeast. Its activation is modulated via phosphorylation at specific sites performed by distinct kinases involved in signal transduction cascades triggered by various nutrients. The TORC1 complex, comprising the regulatory subunits Lst8p, Tco89p, and Kog1p, alongside either Tor1p or Tor2p as the catalytic subunit, primarily responds to amino acid availability. Beyond Sch9, TORC1 targets additional key effectors involved in cell growth and anabolic processes such as PKA and Tap42. The Pkh kinases (Pkhs) are involved in the response to sphingolipids, whereas Bur1p and Pho85p are cyclin-dependent kinases, the latter belonging to the phosphate signaling pathway. Consequently, the activation of Sch9p represses Rim1p5, thereby downregulating the transcription of essential genes required for stress resistance and survival.

**Table 1:** Differences between replicative lifespan (RLS) and chronological lifespan (CLS) in yeast.

	Replicative Lifespan (RLS)	Chronological Lifespan (CLS)
Core Measurement	Number of daughter cells a “mother” cell can produce before senescence.	Survival time of a population of non-dividing cells in a post-mitotic state.
Metabolic State	Primarily glycolysis (glucose-rich media such as YPD are used).	Primarily respiration (high respiratory rate during post-diauxic phase).
Human Tissue Analogy	Models aging in actively dividing cells (e.g., certain stem cells).	Models aging in non-dividing/differentiated tissues (e.g., neurons, muscle).
Manual Method	Micromanipulation (dissection of buds from mother cells).	Growing cells to saturation in liquid synthetic media (SD) followed by incubation and plating every 2 days to count CFUs.
Technological Improvements (examples)	1. Mother Enrichment Program (MEP): Uses Cre-estradiol system to arrest daughter cells. 2. Microfluidics: Traps mother cells by size while flushing daughters away.	1. 96-well Plate Automation: Measures optical density (OD) to screen large libraries. 2. High Throughput: Estimates survival by outgrowth in rich media (YPD).

**Table 2:** Assays used during CLS to monitor genomic instability.

Assay Type	Target/Marker	Mechanism/Methodology
Forward mutation assay	*CAN1* *URA3* *STE2*	Resistance to toxic canavanine; cells grow only if the *CAN1* gene is inactivated by any type of mutation. Resistance to 5-FOA. Cells grow only if *URA3* gene is inactivated Inactivating mutations on *STE2* gene override the cell division block of alfa-factor.
Reverse mutation assay: point mutations	*TRP1* Amber Reversion	Reversion of a specific stop codon (Trp1-289) to restore tryptophan prototrophy (growth on SDC-Trp).
Reverse mutation assay: frameshift mutations	*LYS2* Locus	Restoration of the wild-type reading frame in a specifically engineered *lys2* insertion mutant.
Recombination assay	*HXT13*/*URA3*/*CAN1*	Loss of a 7.5 kb DNA fragment allows survival on medium containing both fluoroorotic acid and canavanine.

## Data Availability

No new data were created or analyzed in this study. Data sharing is not applicable to this article.

## References

[1] McGovern P., Jalabadze M., Batiuk S., Callahan M.P., Smith K.E., Hall G.R., Kvavadze E., Maghradze D., Rusishvili N., Bouby L. (2017). Early Neolithic wine of Georgia in the South Caucasus. Proc. Natl. Acad. Sci. USA.

[2] Xu P., Zhang X., Wang Y., Rahman S.U., Huang D., Wu Z. (2026). Yeast Chronological Lifespan Model as a Tool for Screening Aging Interventions. Int. J. Mol. Sci..

[3] Hamczyk M.R., Nevado R.M., Barettino A., Fuster V., Andrés V. (2020). Biological Versus Chronological Aging: JACC Focus Seminar. J. Am. Coll. Cardiol..

[4] Toniolo L., Sirago G., Giacomello E. (2023). Experimental models for ageing research. Histol. Histopathol..

[5] Cohen A.A. (2018). Aging across the tree of life: The importance of a comparative perspective for the use of animal models in aging. Biochim Biophys. Acta Mol. Basis Dis..

[6] Wei M., Fabrizio P., Hu J., Ge H., Cheng C., Li L., Longo V.D. (2008). Life span extension by calorie restriction depends on Rim15 and transcription factors downstream of Ras/PKA, Tor, and Sch9. PLoS Genet..

[7] David L., Huber W., Granovskaia M., Toedling J., Palm C.J., Bofkin L., Jones T., Davis R.W., Steinmetz L.M. (2006). A high-resolution map of transcription in the yeast genome. Proc. Natl. Acad. Sci. USA.

[8] Michaelis A.C., Brunner A.D., Zwiebel M., Meier F., Strauss M.T., Bludau I., Mann M. (2023). The social and structural architecture of the yeast protein interactome. Nature.

[9] Huh W.K., Falvo J.V., Gerke L.C., Carroll A.S., Howson R.W., Weissman J.S., O’Shea E.K. (2003). Global analysis of protein localization in budding yeast. Nature.

[10] Oftadeh O., Salvy P., Masid M., Curvat M., Miskovic L., Hatzimanikatis V. (2021). A genome-scale metabolic model of *Saccharomyces cerevisiae* that integrates expression constraints and reaction thermodynamics. Nat. Commun..

[11] Laurent J.M., Young J.H., Kachroo A.H., Marcotte E.M. (2016). Efforts to make and apply humanized yeast. Brief. Funct. Genom..

[12] Kachroo A.H., Vandeloo M., Greco B.M., Abdullah M. (2022). Humanized yeast to model human biology, disease and evolution. Dis. Model Mech..

[13] Karathia H., Vilaprinyo E., Sorribas A., Alves R. (2011). *Saccharomyces cerevisiae* as a model organism: A comparative study. PLoS ONE.

[14] Foury F. (1997). Human genetic diseases: A cross-talk between man and yeast. Gene.

[15] Mortimer R.K., Johnston J.R. (1959). Life span of individual yeast cells. Nature.

[16] Postnikoff S.D., Harkness T.A. (2014). Replicative and chronological life-span assays. Methods Mol. Biol..

[17] Chen C., Dewaele S., Braeckman B., Desmyter L., Verstraelen J., Borgonie G., Vanfleteren J., Contreras R. (2003). A high-throughput screening system for genes extending life-span. Exp. Gerontol..

[18] Chen C., Contreras R. (2004). The bud scar-based screening system for hunting human genes extending life span. Ann. N. Y. Acad. Sci..

[19] Lindstrom D.L., Gottschling D.E. (2009). The mother enrichment program: A genetic system for facile replicative life span analysis in *Saccharomyces cerevisiae*. Genetics.

[20] He C., Zhou C., Kennedy B.K. (2018). The yeast replicative aging model. Biochim. Biophys. Acta Mol. Basis Dis..

[21] Chen K.L., Crane M.M., Kaeberlein M. (2017). Microfluidic technologies for yeast replicative lifespan studies. Mech. Ageing Dev..

[22] Wang Y., Zhu Z., Liu K., Xiao Q., Geng Y., Xu F., Ouyang S., Zheng K., Fan Y., Jin N. (2022). A high-throughput microfluidic diploid yeast long-term culturing (DYLC) chip capable of bud reorientation and concerted daughter dissection for replicative lifespan determination. J. Nanobiotechnol..

[23] Botta G., Turn C.S., Quintyne N.J., Kirchman P.A. (2011). Increased iron supplied through Fet3p results in replicative life span extension of *Saccharomyces cerevisiae* under conditions requiring respiratory metabolism. Exp. Gerontol..

[24] Kaya A., Ma S., Wasko B., Lee M., Kaeberlein M., Gladyshev V.N. (2015). Defining Molecular Basis for Longevity Traits in Natural Yeast Isolates. npj Aging Mech. Dis..

[25] Hendrickson D.G., Soifer I., Wranik B.J., Kim G., Robles M., Gibney P.A., McIsaac R.S. (2018). A new experimental platform facilitates assessment of the transcriptional and chromatin landscapes of aging yeast. eLife.

[26] Leupold S., Hubmann G., Litsios A., Meinema A.C., Takhaveev V., Papagiannakis A., Niebel B., Janssens G., Siegel D., Heinemann M. (2019). *Saccharomyces cerevisiae* goes through distinct metabolic phases during its replicative lifespan. eLife.

[27] Kirchman P.A., Kim S., Lai C.Y., Jazwinski S.M. (1999). Interorganelle signaling is a determinant of longevity in *Saccharomyces cerevisiae*. Genetics.

[28] Giannattasio S., Guaragnella N., Zdralević M., Marra E. (2013). Molecular mechanisms of *Saccharomyces cerevisiae* stress adaptation and programmed cell death in response to acetic acid. Front. Microbiol..

[29] Longo V.D., Ellerby L.M., Bredesen D.E., Valentine J.S., Gralla E.B. (1997). Human Bcl-2 reverses survival defects in yeast lacking superoxide dismutase and delays death of wild-type yeast. J. Cell Biol..

[30] Longo V.D., Gralla E.B., Valentine J.S. (1996). Superoxide dismutase activity is essential for stationary phase survival in *Saccharomyces cerevisiae*. Mitochondrial production of toxic oxygen species in vivo. J. Biol. Chem..

[31] Mirisola M.G., Longo V.D. (2022). Yeast Chronological Lifespan: Longevity Regulatory Genes and Mechanisms. Cells.

[32] Powers R.W., Kaeberlein M., Caldwell S.D., Kennedy B.K., Fields S. (2006). Extension of chronological life span in yeast by decreased TOR pathway signaling. Genes Dev..

[33] Deprez M.A., Eskes E., Winderickx J., Wilms T. (2018). The TORC1-Sch9 pathway as a crucial mediator of chronological lifespan in the yeast *Saccharomyces cerevisiae*. FEMS Yeast Res..

[34] Ocampo A., Barrientos A. (2011). Quick and reliable assessment of chronological life span in yeast cell populations by flow cytometry. Mech. Ageing Dev..

[35] Hayles J., Nurse P. (2018). Introduction to Fission Yeast as a Model System. Cold Spring Harb. Protoc..

[36] Erjavec N., Cvijovic M., Klipp E., Nyström T. (2008). Selective benefits of damage partitioning in unicellular systems and its effects on aging. Proc. Natl. Acad. Sci. USA.

[37] Spivey E.C., Jones S.K., Rybarski J.R., Saifuddin F.A., Finkelstein I.J. (2017). An aging-independent replicative lifespan in a symmetrically dividing eukaryote. eLife.

[38] Ohtsuka H., Shimasaki T., Aiba H. (2021). Genes affecting the extension of chronological lifespan in Schizosaccharomyces pombe (fission yeast). Mol. Microbiol..

[39] Werner-Washburne M., Braun E.L., Crawford M.E., Peck V.M. (1996). Stationary phase in *Saccharomyces cerevisiae*. Mol. Microbiol..

[40] Hu J., Wei M., Mirisola M.G., Longo V.D. (2013). Assessing chronological aging in *Saccharomyces cerevisiae*. Methods Mol. Biol..

[41] Kaeberlein M., Powers R.W. (2007). Sir2 and calorie restriction in yeast: A skeptical perspective. Ageing Res. Rev..

[42] Huberts D.H., González J., Lee S.S., Litsios A., Hubmann G., Wit E.C., Heinemann M. (2014). Calorie restriction does not elicit a robust extension of replicative lifespan in *Saccharomyces cerevisiae*. Proc. Natl. Acad. Sci. USA.

[43] Albertyn J., Hohmann S., Thevelein J.M., Prior B.A. (1994). GPD1, which encodes glycerol-3-phosphate dehydrogenase, is essential for growth under osmotic stress in *Saccharomyces cerevisiae*, and its expression is regulated by the high-osmolarity glycerol response pathway. Mol. Cell. Biol..

[44] Nakaoka H., Wakamoto Y. (2017). Aging, mortality, and the fast growth trade-off of Schizosaccharomyces pombe. PLoS Biol..

[45] Mirisola M.G., Longo V.D. (2012). Acetic acid and acidification accelerate chronological and replicative aging in yeast. Cell Cycle.

[46] Murakami C., Delaney J.R., Chou A., Carr D., Schleit J., Sutphin G.L., An E.H., Castanza A.S., Fletcher M., Goswami S. (2012). PH neutralization protects against reduction in replicative lifespan following chronological aging in yeast. Cell Cycle.

[47] Mirisola M.G., Braun R.J., Petranovic D. (2014). Approaches to study yeast cell aging and death. FEMS Yeast Res..

[48] Ocampo A., Liu J., Schroeder E.A., Shadel G.S., Barrientos A. (2012). Mitochondrial respiratory thresholds regulate yeast chronological life span and its extension by caloric restriction. Cell Metab..

[49] Burtner C.R., Murakami C.J., Kennedy B.K., Kaeberlein M. (2009). A molecular mechanism of chronological aging in yeast. Cell Cycle.

[50] Wasko B.M., Carr D.T., Tung H., Doan H., Schurman N., Neault J.R., Feng J., Lee J., Zipkin B., Mouser J. (2013). Buffering the pH of the culture medium does not extend yeast replicative lifespan. F1000Research.

[51] Semchyshyn H.M., Abrat O.B., Miedzobrodzki J., Inoue Y., Lushchak V.I. (2011). Acetate but not propionate induces oxidative stress in bakers’ yeast *Saccharomyces cerevisiae*. Redox Rep..

[52] Kitanovic A., Bonowski F., Heigwer F., Ruoff P., Kitanovic I., Ungewiss C., Wölfl S. (2012). Acetic acid treatment in S. cerevisiae creates significant energy deficiency and nutrient starvation that is dependent on the activity of the mitochondrial transcriptional complex Hap2-3-4-5. Front. Oncol..

[53] Colombo S., Ma P., Cauwenberg L., Winderickx J., Crauwels M., Teunissen A., Nauwelaers D., de Winde J.H., Gorwa M.F., Colavizza D. (1998). Involvement of distinct G-proteins, Gpa2 and Ras, in glucose- and intracellular acidification-induced cAMP signalling in the yeast *Saccharomyces cerevisiae*. EMBO J..

[54] Burhans W.C., Weinberger M. (2009). Acetic acid effects on aging in budding yeast: Are they relevant to aging in higher eukaryotes?. Cell Cycle.

[55] Leontieva O.V., Blagosklonny M.V. (2011). Yeast-like chronological senescence in mammalian cells: Phenomenon, mechanism and pharmacological suppression. Aging.

[56] Fabrizio P., Battistella L., Vardavas R., Gattazzo C., Liou L.L., Diaspro A., Dossen J.W., Gralla E.B., Longo V.D. (2004). Superoxide is a mediator of an altruistic aging program in *Saccharomyces cerevisiae*. J. Cell Biol..

[57] Hohmann S. (2002). Osmotic stress signaling and osmoadaptation in yeasts. Microbiol. Mol. Biol. Rev..

[58] Mirisola M.G., Taormina G., Fabrizio P., Wei M., Hu J., Longo V.D. (2014). Serine- and threonine/valine-dependent activation of PDK and Tor orthologs converge on Sch9 to promote aging. PLoS Genet..

[59] Mohammad K., Baratang Junio J.A., Tafakori T., Orfanos E., Titorenko V.I. (2020). Mechanisms that Link Chronological Aging to Cellular Quiescence in Budding Yeast. Int. J. Mol. Sci..

[60] López-Otín C., Blasco M.A., Partridge L., Serrano M., Kroemer G. (2013). The hallmarks of aging. Cell.

[61] López-Otín C., Blasco M.A., Partridge L., Serrano M., Kroemer G. (2023). Hallmarks of aging: An expanding universe. Cell.

[62] Hanahan D., Weinberg R.A. (2011). Hallmarks of cancer: The next generation. Cell.

[63] Cagan A., Baez-Ortega A., Brzozowska N., Abascal F., Coorens T.H.H., Sanders M.A., Lawson A.R.J., Harvey L.M.R., Bhosle S., Jones D. (2022). Somatic mutation rates scale with lifespan across mammals. Nature.

[64] Gillooly J.F., Allen A.P., West G.B., Brown J.H. (2005). The rate of DNA evolution: Effects of body size and temperature on the molecular clock. Proc. Natl. Acad. Sci. USA.

[65] Abascal F., Harvey L.M.R., Mitchell E., Lawson A.R.J., Lensing S.V., Ellis P., Russell A.J.C., Alcantara R.E., Baez-Ortega A., Wang Y. (2021). Somatic mutation landscapes at single-molecule resolution. Nature.

[66] Madia F., Gattazzo C., Fabrizio P., Longo V.D. (2007). A simple model system for age-dependent DNA damage and cancer. Mech. Ageing Dev..

[67] Lang G.I., Murray A.W. (2008). Estimating the per-base-pair mutation rate in the yeast *Saccharomyces cerevisiae*. Genetics.

[68] Capizzi R.L., Jameson J.W. (1973). A table for the estimation of the spontaneous mutation rate of cells in culture. Mutat. Res..

[69] Chen C., Umezu K., Kolodner R.D. (1998). Chromosomal rearrangements occur in S. cerevisiae rfa1 mutator mutants due to mutagenic lesions processed by double-strand-break repair. Mol. Cell.

[70] Zhu Y.O., Siegal M.L., Hall D.W., Petrov D.A. (2014). Precise estimates of mutation rate and spectrum in yeast. Proc. Natl. Acad. Sci. USA.

[71] Chen J.B., Sun J., Jazwinski S.M. (1990). Prolongation of the yeast life span by the v-Ha-RAS oncogene. Mol. Microbiol..

[72] Campisi J., d’Adda di Fagagna F. (2007). Cellular senescence: When bad things happen to good cells. Nat. Rev. Mol. Cell Biol..

[73] Sun J., Kale S.P., Childress A.M., Pinswasdi C., Jazwinski S.M. (1994). Divergent roles of RAS1 and RAS2 in yeast longevity. J. Biol. Chem..

[74] Chen C., Rodriguez Pino M., Haller P.R., Verde F. (2019). Conserved NDR/LATS kinase controls RAS GTPase activity to regulate cell growth and chronological lifespan. Mol. Biol. Cell.

[75] Longo V.D. (2003). The Ras and Sch9 pathways regulate stress resistance and longevity. Exp. Gerontol..

[76] Longo V.D. (1999). Mutations in signal transduction proteins increase stress resistance and longevity in yeast, nematodes, fruit flies, and mammalian neuronal cells. Neurobiol. Aging.

[77] Downward J. (1997). Cell cycle: Routine role for Ras. Curr. Biol..

[78] Marshall C.J. (1996). Ras effectors. Curr. Opin. Cell Biol..

[79] McCormick F. (1995). Ras-related proteins in signal transduction and growth control. Mol. Reprod. Dev..

[80] Roux A.E., Quissac A., Chartrand P., Ferbeyre G., Rokeach L.A. (2006). Regulation of chronological aging in Schizosaccharomyces pombe by the protein kinases Pka1 and Sck2. Aging Cell.

[81] Longo V.D., Fabrizio P. (2012). Chronological aging in *Saccharomyces cerevisiae*. Subcell. Biochem..

[82] Creamer D.R., Hubbard S.J., Ashe M.P., Grant C.M. (2022). Yeast Protein Kinase A Isoforms: A Means of Encoding Specificity in the Response to Diverse Stress Conditions?. Biomolecules.

[83] Fabrizio P., Liou L.L., Moy V.N., Diaspro A., Valentine J.S., Gralla E.B., Longo V.D. (2003). SOD2 functions downstream of Sch9 to extend longevity in yeast. Genetics.

[84] Pedruzzi I., Bürckert N., Egger P., De Virgilio C. (2000). *Saccharomyces cerevisiae* Ras/cAMP pathway controls post-diauxic shift element-dependent transcription through the zinc finger protein Gis1. EMBO J..

[85] Liu Y., Yang F., Li S., Dai J., Deng H. (2018). Glutaredoxin Deletion Shortens Chronological Life Span in *Saccharomyces cerevisiae* via ROS-Mediated Ras/PKA Activation. J. Proteome Res..

[86] Martínez-Pastor M.T., Marchler G., Schüller C., Marchler-Bauer A., Ruis H., Estruch F. (1996). The *Saccharomyces cerevisiae* zinc finger proteins Msn2p and Msn4p are required for transcriptional induction through the stress response element (STRE). EMBO J..

[87] Longo V.D. (1997). The Chronological Life Span of *Saccharomyces cerevisiae*. Studies of Superoxide Dismutase, Ras and Bcl-2. Ph.D. Thesis.

[88] Hibi T., Ohtsuka H., Shimasaki T., Inui S., Shibuya M., Tatsukawa H., Kanie K., Yamamoto Y., Aiba H. (2018). Tschimganine and its derivatives extend the chronological life span of yeast via activation of the Sty1 pathway. Genes Cells.

[89] Hu Y., Luo Y., Yin D., Zhao L., Wang Y., Yao R., Zhang P., Wu X., Li M., Hidalgo E. (2022). Schizosaccharomyces pombe MAP kinase Sty1 promotes survival of Δppr10 cells with defective mitochondrial protein synthesis. Int. J. Biochem Cell Biol..

[90] Zuin A., Carmona M., Morales-Ivorra I., Gabrielli N., Vivancos A.P., Ayté J., Hidalgo E. (2010). Lifespan extension by calorie restriction relies on the Sty1 MAP kinase stress pathway. EMBO J..

[91] Roux A.E., Leroux A., Alaamery M.A., Hoffman C.S., Chartrand P., Ferbeyre G., Rokeach L.A. (2009). Pro-aging effects of glucose signaling through a G protein-coupled glucose receptor in fission yeast. PLoS Genet..

[92] Enns L.C., Morton J.F., Mangalindan R.S., McKnight G.S., Schwartz M.W., Kaeberlein M.R., Kennedy B.K., Rabinovitch P.S., Ladiges W.C. (2009). Attenuation of age-related metabolic dysfunction in mice with a targeted disruption of the Cbeta subunit of protein kinase A. J. Gerontol. A Biol. Sci. Med. Sci..

[93] Enns L.C., Morton J.F., Treuting P.R., Emond M.J., Wolf N.S., Dai D.F., McKnight G.S., Rabinovitch P.S., Ladiges W.C. (2009). Disruption of protein kinase A in mice enhances healthy aging. PLoS ONE.

[94] Enns L.C., Ladiges W. (2010). Protein kinase A signaling as an anti-aging target. Ageing Res. Rev..

[95] Yan L., Vatner D.E., O’Connor J.P., Ivessa A., Ge H., Chen W., Hirotani S., Ishikawa Y., Sadoshima J., Vatner S.F. (2007). Type 5 adenylyl cyclase disruption increases longevity and protects against stress. Cell.

[96] Sehgal S.N., Baker H., Vézina C. (1975). Rapamycin (AY-22,989), a new antifungal antibiotic. II. Fermentation, isolation and characterization. J. Antibiot..

[97] Bonawitz N.D., Chatenay-Lapointe M., Pan Y., Shadel G.S. (2007). Reduced TOR signaling extends chronological life span via increased respiration and upregulation of mitochondrial gene expression. Cell Metab..

[98] Kaeberlein M., Powers R.W., Steffen K.K., Westman E.A., Hu D., Dang N., Kerr E.O., Kirkland K.T., Fields S., Kennedy B.K. (2005). Regulation of yeast replicative life span by TOR and Sch9 in response to nutrients. Science.

[99] Longo V.D., Liou L.L., Valentine J.S., Gralla E.B. (1999). Mitochondrial superoxide decreases yeast survival in stationary phase. Arch. Biochem. Biophys..

[100] Loewith R., Jacinto E., Wullschleger S., Lorberg A., Crespo J.L., Bonenfant D., Oppliger W., Jenoe P., Hall M.N. (2002). Two TOR complexes, only one of which is rapamycin sensitive, have distinct roles in cell growth control. Mol. Cell.

[101] Lerner C., Bitto A., Pulliam D., Nacarelli T., Konigsberg M., Van Remmen H., Torres C., Sell C. (2013). Reduced mammalian target of rapamycin activity facilitates mitochondrial retrograde signaling and increases life span in normal human fibroblasts. Aging Cell.

[102] Morozumi Y., Shiozaki K. (2021). Conserved and Divergent Mechanisms That Control TORC1 in Yeasts and Mammals. Genes.

[103] Albig A.R., Decker C.J. (2001). The target of rapamycin signaling pathway regulates mRNA turnover in the yeast *Saccharomyces cerevisiae*. Mol. Biol. Cell.

[104] Stracka D., Jozefczuk S., Rudroff F., Sauer U., Hall M.N. (2014). Nitrogen source activates TOR (target of rapamycin) complex 1 via glutamine and independently of Gtr/Rag proteins. J. Biol. Chem..

[105] Wullschleger S., Loewith R., Hall M.N. (2006). TOR signaling in growth and metabolism. Cell.

[106] Zhang T., Péli-Gulli M.P., Yang H., De Virgilio C., Ding J. (2012). Ego3 functions as a homodimer to mediate the interaction between Gtr1-Gtr2 and Ego1 in the ego complex to activate TORC1. Structure.

[107] Cecil J.H., Padilla C.M., Lipinski A.A., Langlais P.R., Luo X., Capaldi A.P. (2025). The Molecular Logic of Gtr1/2 and Pib2 Dependent TORC1 Regulation in Budding Yeast. bioRxiv.

[108] Péli-Gulli M.P., Raucci S., Hu Z., Dengjel J., De Virgilio C. (2017). Feedback Inhibition of the Rag GTPase GAP Complex Lst4-Lst7 Safeguards TORC1 from Hyperactivation by Amino Acid Signals. Cell Rep..

[109] Troutman K.K., Varlakhanova N.V., Tornabene B.A., Ramachandran R., Ford M.G.J. (2022). Conserved Pib2 regions have distinct roles in TORC1 regulation at the vacuole. J. Cell Sci..

[110] Yuan W., Guo S., Gao J., Zhong M., Yan G., Wu W., Chao Y., Jiang Y. (2017). General Control Nonderepressible 2 (GCN2) Kinase Inhibits Target of Rapamycin Complex 1 in Response to Amino Acid Starvation in. J. Biol. Chem..

[111] Santhanam A., Hartley A., Düvel K., Broach J.R., Garrett S. (2004). PP2A phosphatase activity is required for stress and Tor kinase regulation of yeast stress response factor Msn2p. Eukaryot. Cell.

[112] Li J., Yan G., Liu S., Jiang T., Zhong M., Yuan W., Chen S., Zheng Y., Jiang Y., Jiang Y. (2017). Target of rapamycin complex 1 and Tap42-associated phosphatases are required for sensing changes in nitrogen conditions in the yeast *Saccharomyces cerevisiae*. Mol. Microbiol..

[113] Jiang Y., Broach J.R. (1999). Tor proteins and protein phosphatase 2A reciprocally regulate Tap42 in controlling cell growth in yeast. EMBO J..

[114] Wang H., Jiang Y. (2003). The Tap42-protein phosphatase type 2A catalytic subunit complex is required for cell cycle-dependent distribution of actin in yeast. Mol. Cell. Biol..

[115] Yorimitsu T., He C., Wang K., Klionsky D.J. (2009). Tap42-associated protein phosphatase type 2A negatively regulates induction of autophagy. Autophagy.

[116] Düvel K., Santhanam A., Garrett S., Schneper L., Broach J.R. (2003). Multiple roles of Tap42 in mediating rapamycin-induced transcriptional changes in yeast. Mol. Cell.

[117] Yan G., Shen X., Jiang Y. (2006). Rapamycin activates Tap42-associated phosphatases by abrogating their association with Tor complex 1. EMBO J..

[118] Rodríguez-López M., Gonzalez S., Hillson O., Tunnacliffe E., Codlin S., Tallada V.A., Bähler J., Rallis C. (2020). The GATA Transcription Factor Gaf1 Represses tRNAs, Inhibits Growth, and Extends Chronological Lifespan Downstream of Fission Yeast TORC1. Cell Rep..

[119] Matsuo T., Otsubo Y., Urano J., Tamanoi F., Yamamoto M. (2007). Loss of the TOR kinase Tor2 mimics nitrogen starvation and activates the sexual development pathway in fission yeast. Mol. Cell. Biol..

[120] Alao J.P., Legon L., Dabrowska A., Tricolici A.M., Kumar J., Rallis C. (2023). Interplays of AMPK and TOR in Autophagy Regulation in Yeast. Cells.

[121] Ohtsuka H., Shimasaki T., Aiba H. (2021). Extension of chronological lifespan in Schizosaccharomyces pombe. Genes Cells.

[122] Fayyadkazan M., Tate J.J., Vierendeels F., Cooper T.G., Dubois E., Georis I. (2014). Components of Golgi-to-vacuole trafficking are required for nitrogen- and TORC1-responsive regulation of the yeast GATA factors. Microbiologyopen.

[123] Liu X.M., Sun L.L., Hu W., Ding Y.H., Dong M.Q., Du L.L. (2015). ESCRTs Cooperate with a Selective Autophagy Receptor to Mediate Vacuolar Targeting of Soluble Cargos. Mol. Cell.

[124] Fabrizio P., Pozza F., Pletcher S.D., Gendron C.M., Longo V.D. (2001). Regulation of longevity and stress resistance by Sch9 in yeast. Science.

[125] Huber A., French S.L., Tekotte H., Yerlikaya S., Stahl M., Perepelkina M.P., Tyers M., Rougemont J., Beyer A.L., Loewith R. (2011). Sch9 regulates ribosome biogenesis via Stb3, Dot6 and Tod6 and the histone deacetylase complex RPD3L. EMBO J..

[126] Swinnen E., Wilms T., Idkowiak-Baldys J., Smets B., De Snijder P., Accardo S., Ghillebert R., Thevissen K., Cammue B., De Vos D. (2014). The protein kinase Sch9 is a key regulator of sphingolipid metabolism in *Saccharomyces cerevisiae*. Mol. Biol. Cell.

[127] Roosen J., Engelen K., Marchal K., Mathys J., Griffioen G., Cameroni E., Thevelein J.M., De Virgilio C., De Moor B., Winderickx J. (2005). PKA and Sch9 control a molecular switch important for the proper adaptation to nutrient availability. Mol. Microbiol..

[128] Toda T., Cameron S., Sass P., Wigler M. (1988). SCH9, a gene of *Saccharomyces cerevisiae* that encodes a protein distinct from, but functionally and structurally related to, cAMP-dependent protein kinase catalytic subunits. Genes Dev..

[129] Novarina D., Guerra P., Milias-Argeitis A. (2021). Vacuolar Localization via the N-terminal Domain of Sch9 is Required for TORC1-dependent Phosphorylation and Downstream Signal Transduction. J. Mol. Biol..

[130] Voordeckers K., Kimpe M., Haesendonckx S., Louwet W., Versele M., Thevelein J.M. (2011). Yeast 3-phosphoinositide-dependent protein kinase-1 (PDK1) orthologs Pkh1-3 differentially regulate phosphorylation of protein kinase A (PKA) and the protein kinase B (PKB)/S6K ortholog Sch9. J. Biol. Chem..

[131] Wilms T., Swinnen E., Eskes E., Dolz-Edo L., Uwineza A., Van Essche R., Rosseels J., Zabrocki P., Cameroni E., Franssens V. (2017). The yeast protein kinase Sch9 adjusts V-ATPase assembly/disassembly to control pH homeostasis and longevity in response to glucose availability. PLoS Genet..

[132] Toker A., Newton A.C. (2000). Cellular signaling: Pivoting around PDK-1. Cell.

[133] Huang X., Liu J., Dickson R.C. (2012). Down-regulating sphingolipid synthesis increases yeast lifespan. PLoS Genet..

[134] Urban J., Soulard A., Huber A., Lippman S., Mukhopadhyay D., Deloche O., Wanke V., Anrather D., Ammerer G., Riezman H. (2007). Sch9 is a major target of TORC1 in *Saccharomyces cerevisiae*. Mol. Cell.

[135] Jin Y., Jin N., Oikawa Y., Benyair R., Koizumi M., Wilson T.E., Ohsumi Y., Weisman L.S. (2022). Bur1 functions with TORC1 for vacuole-mediated cell cycle progression. EMBO Rep..

[136] Lenburg M.E., O’Shea E.K. (1996). Signaling phosphate starvation. Trends Biochem. Sci..

[137] Deprez M.A., Caligaris M., Rosseels J., Hatakeyama R., Ghillebert R., Sampaio-Marques B., Mudholkar K., Eskes E., Meert E., Ungermann C. (2023). The nutrient-responsive CDK Pho85 primes the Sch9 kinase for its activation by TORC1. PLoS Genet..

[138] Lee M., O’Regan S., Moreau J.L., Johnson A.L., Johnston L.H., Goding C.R. (2000). Regulation of the Pcl7-Pho85 cyclin-cdk complex by Pho81. Mol. Microbiol..

[139] Mouillon J.M., Persson B.L. (2006). New aspects on phosphate sensing and signalling in *Saccharomyces cerevisiae*. FEMS Yeast Res..

[140] Nakashima A., Otsubo Y., Yamashita A., Sato T., Yamamoto M., Tamanoi F. (2012). Psk1, an AGC kinase family member in fission yeast, is directly phosphorylated and controlled by TORC1 and functions as S6 kinase. J. Cell Sci..

[141] Zuin A., Castellano-Esteve D., Ayté J., Hidalgo E. (2010). Living on the edge: Stress and activation of stress responses promote lifespan extension. Aging.

[142] Caligaris M., Nicastro R., Hu Z., Tripodi F., Hummel J.E., Pillet B., Deprez M.A., Winderickx J., Rospert S., Coccetti P. (2023). Snf1/AMPK fine-tunes TORC1 signaling in response to glucose starvation. eLife.

[143] Coccetti P., Nicastro R., Tripodi F. (2018). Conventional and emerging roles of the energy sensor Snf1/AMPK in *Saccharomyces cerevisiae*. Microb. Cell.

[144] Zhang A., Shen Y., Gao W., Dong J. (2011). Role of Sch9 in regulating Ras-cAMP signal pathway in *Saccharomyces cerevisiae*. FEBS Lett..

[145] Madia F., Wei M., Yuan V., Hu J., Gattazzo C., Pham P., Goodman M.F., Longo V.D. (2009). Oncogene homologue Sch9 promotes age-dependent mutations by a superoxide and Rev1/Polzeta-dependent mechanism. J. Cell Biol..

[146] Lavoie H., Whiteway M. (2008). Increased respiration in the sch9Delta mutant is required for increasing chronological life span but not replicative life span. Eukaryot. Cell.

[147] Ludovico P., Burhans W.C. (2014). Reactive oxygen species, ageing and the hormesis police. FEMS Yeast Res..

[148] Williams T.D., Joshua I., Soubigou F., Dublanska S.M., Bergquist R., Rousseau A. (2024). Distinct TORC1 signalling branches regulate Adc17 proteasome assembly chaperone expression. J. Cell Sci..

[149] Montella-Manuel S., Pujol-Carrion N., de la Torre-Ruiz M.A. (2021). The Cell Wall Integrity Receptor Mtl1 Contributes to Articulate Autophagic Responses When Glucose Availability Is Compromised. J. Fungi.

[150] Fontana L., Partridge L., Longo V.D. (2010). Extending healthy life span--from yeast to humans. Science.

[151] Kapahi P., Kaeberlein M., Hansen M. (2017). Dietary restriction and lifespan: Lessons from invertebrate models. Ageing Res. Rev..

[152] Lushchak O., Strilbytska O., Piskovatska V., Storey K.B., Koliada A., Vaiserman A. (2017). The role of the TOR pathway in mediating the link between nutrition and longevity. Mech. Ageing Dev..

[153] Hara K., Yonezawa K., Weng Q.P., Kozlowski M.T., Belham C., Avruch J. (1998). Amino acid sufficiency and mTOR regulate p70 S6 kinase and eIF-4E BP1 through a common effector mechanism. J. Biol. Chem..

[154] Vellai T., Takacs-Vellai K., Zhang Y., Kovacs A.L., Orosz L., Müller F. (2003). Genetics: Influence of TOR kinase on lifespan in C. elegans. Nature.

[155] Selman C., Tullet J.M., Wieser D., Irvine E., Lingard S.J., Choudhury A.I., Claret M., Al-Qassab H., Carmignac D., Ramadani F. (2009). Ribosomal protein S6 kinase 1 signaling regulates mammalian life span. Science.

[156] Zid B.M., Rogers A.N., Katewa S.D., Vargas M.A., Kolipinski M.C., Lu T.A., Benzer S., Kapahi P. (2009). 4E-BP extends lifespan upon dietary restriction by enhancing mitochondrial activity in Drosophila. Cell.

[157] Gao X., Zhang Y., Arrazola P., Hino O., Kobayashi T., Yeung R.S., Ru B., Pan D. (2002). Tsc tumour suppressor proteins antagonize amino-acid-TOR signalling. Nat. Cell Biol..

[158] Kapahi P., Zid B.M., Harper T., Koslover D., Sapin V., Benzer S. (2004). Regulation of lifespan in Drosophila by modulation of genes in the TOR signaling pathway. Curr. Biol..

[159] Harrison D.E., Strong R., Sharp Z.D., Nelson J.F., Astle C.M., Flurkey K., Nadon N.L., Wilkinson J.E., Frenkel K., Carter C.S. (2009). Rapamycin fed late in life extends lifespan in genetically heterogeneous mice. Nature.

[160] Hurez V., Dao V., Liu A., Pandeswara S., Gelfond J., Sun L., Bergman M., Orihuela C.J., Galvan V., Padrón Á. (2015). Chronic mTOR inhibition in mice with rapamycin alters T, B, myeloid, and innate lymphoid cells and gut flora and prolongs life of immune-deficient mice. Aging Cell.

[161] Zhou X.L., Xu J.J., Ni Y.H., Chen X.C., Zhang H.X., Zhang X.M., Liu W.J., Luo L.L., Fu Y.C. (2014). SIRT1 activator (SRT1720) improves the follicle reserve and prolongs the ovarian lifespan of diet-induced obesity in female mice via activating SIRT1 and suppressing mTOR signaling. J. Ovarian Res..

[162] Zhang X.M., Li L., Xu J.J., Wang N., Liu W.J., Lin X.H., Fu Y.C., Luo L.L. (2013). Rapamycin preserves the follicle pool reserve and prolongs the ovarian lifespan of female rats via modulating mTOR activation and sirtuin expression. Gene.

[163] Gallage S., Irvine E.E., Barragan Avila J.E., Reen V., Pedroni S.M.A., Duran I., Ranvir V., Khadayate S., Pombo J., Brookes S. (2024). Ribosomal S6 kinase 1 regulates inflammaging via the senescence secretome. Nat. Aging.

[164] Lee P., Cho B.R., Joo H.S., Hahn J.S. (2008). Yeast Yak1 kinase, a bridge between PKA and stress-responsive transcription factors, Hsf1 and Msn2/Msn4. Mol. Microbiol..

[165] Medvedik O., Lamming D.W., Kim K.D., Sinclair D.A. (2007). MSN2 and MSN4 link calorie restriction and TOR to sirtuin-mediated lifespan extension in *Saccharomyces cerevisiae*. PLoS Biol..

[166] Marchler G., Schüller C., Adam G., Ruis H. (1993). A *Saccharomyces cerevisiae* UAS element controlled by protein kinase A activates transcription in response to a variety of stress conditions. EMBO J..

[167] Pascual-Ahuir A., Proft M. (2007). Control of stress-regulated gene expression and longevity by the Sch9 protein kinase. Cell Cycle.

[168] Fiorito G., Tosti V., Polidoro S., Bertozzi B., Veronese N., Cava E., Spelta F., Piccio L., Early D.S., Raftery D. (2025). Multi-omic analysis of biological aging biomarkers in long-term calorie restriction and endurance exercise practitioners: A cross-sectional study. Aging Cell.

[169] Cantó C., Auwerx J. (2009). Caloric restriction, SIRT1 and longevity. Trends Endocrinol. Metab..

[170] Most J., Tosti V., Redman L.M., Fontana L. (2017). Calorie restriction in humans: An update. Ageing Res. Rev..

[171] Levine M.E., Suarez J.A., Brandhorst S., Balasubramanian P., Cheng C.W., Madia F., Fontana L., Mirisola M.G., Guevara-Aguirre J., Wan J. (2014). Low protein intake is associated with a major reduction in IGF-1, cancer, and overall mortality in the 65 and younger but not older population. Cell Metab..

[172] Herker E., Jungwirth H., Lehmann K.A., Maldener C., Fröhlich K.U., Wissing S., Büttner S., Fehr M., Sigrist S., Madeo F. (2004). Chronological aging leads to apoptosis in yeast. J. Cell Biol..

[173] Flattery-O’Brien J.A., Grant C.M., Dawes I.W. (1997). Stationary-phase regulation of the *Saccharomyces cerevisiae* SOD2 gene is dependent on additive effects of HAP2/3/4/5- and STRE-binding elements. Mol. Microbiol..

[174] Campos S.E., Avelar-Rivas J.A., Garay E., Juárez-Reyes A., DeLuna A. (2018). Genomewide mechanisms of chronological longevity by dietary restriction in budding yeast. Aging Cell.

[175] Kaya A., Phua C.Z.J., Lee M., Wang L., Tyshkovskiy A., Ma S., Barre B., Liu W., Harrison B.R., Zhao X. (2021). Evolution of natural lifespan variation and molecular strategies of extended lifespan in yeast. eLife.

[176] Stephan J.S., Yeh Y.Y., Ramachandran V., Deminoff S.J., Herman P.K. (2010). The Tor and cAMP-dependent protein kinase signaling pathways coordinately control autophagy in *Saccharomyces cerevisiae*. Autophagy.

[177] Huang H., Kawamata T., Horie T., Tsugawa H., Nakayama Y., Ohsumi Y., Fukusaki E. (2015). Bulk RNA degradation by nitrogen starvation-induced autophagy in yeast. EMBO J..

[178] Suzuki S.W., Onodera J., Ohsumi Y. (2011). Starvation induced cell death in autophagy-defective yeast mutants is caused by mitochondria dysfunction. PLoS ONE.

[179] Wei M., Fabrizio P., Madia F., Hu J., Ge H., Li L.M., Longo V.D. (2009). Tor1/Sch9-regulated carbon source substitution is as effective as calorie restriction in life span extension. PLoS Genet..

[180] Zhang Y., Naaz A., Cheng T.Y.N., Lin J.J., Gao M., Dorajoo R., Alfatah M. (2025). Systematic transcriptomics analysis of calorie restriction and rapamycin unveils their synergistic interaction in prolonging cellular lifespan. Commun. Biol..

[181] Kim J., Kundu M., Viollet B., Guan K.L. (2011). AMPK and mTOR regulate autophagy through direct phosphorylation of Ulk1. Nat. Cell Biol..

[182] Bhattacharya S., Sutter B.M., Tu B.P. (2026). Yeast Rpd3L histone deacetylase couples nutrient shifts to genome-wide chromatin reprogramming. Cell Rep..

[183] Soukar I., Mitra A., Vo L., Rofoo M., Greenberg M.L., Pile L.A. (2025). The histone modification regulator, SIN3, plays a role in the cellular response to changes in glycolytic flux. PLoS ONE.

[184] Caraba B., Stirpe M., Palermo V., Ayala Alban A., Montanari A., Bianchi M.M., Falcone C., Mazzoni C. (2026). Calorie Restriction Suppresses Premature Ageing in Pro-Apoptotic Yeast Mutants Through an Autophagy-Independent Mechanism. Int. J. Mol. Sci..

[185] Uvdal P., Shashkova S. (2023). The Effect of Calorie Restriction on Protein Quality Control in Yeast. Biomolecules.

[186] Schmiedhofer V., Sommersguter-Wagner J., Knittelfelder O., Jungwirth H., Rechberger G.N., Carmona-Gutierrez D., Rockenfeller P., Ruckenstuhl C., Madeo F. (2025). Sugar accelerates chronological aging in yeast via ceramides. Cell Stress.

[187] Mohammad K., Orfanos E., Titorenko V.I. (2021). Caloric restriction causes a distinct reorganization of the lipidome in quiescent and non-quiescent cells of budding yeast. Oncotarget.

[188] Black A., Williams T.D., Soubigou F., Joshua I.M., Zhou H., Lamoliatte F., Rousseau A. (2023). The ribosome-associated chaperone Zuo1 controls translation upon TORC1 inhibition. EMBO J..

[189] Steffen K.K., MacKay V.L., Kerr E.O., Tsuchiya M., Hu D., Fox L.A., Dang N., Johnston E.D., Oakes J.A., Tchao B.N. (2008). Yeast life span extension by depletion of 60s ribosomal subunits is mediated by Gcn4. Cell.

[190] Filer D., Thompson M.A., Takhaveev V., Dobson A.J., Kotronaki I., Green J.W.M., Heinemann M., Tullet J.M.A., Alic N. (2017). RNA polymerase III limits longevity downstream of TORC1. Nature.

[191] Laughery M.F., Wyrick J.J. (2019). Simple CRISPR-Cas9 Genome Editing in *Saccharomyces cerevisiae*. Curr. Protoc. Mol. Biol..

[192] Ozaki Y., Ozaki T., Zhang-Akiyama Q.M. (2026). Glucose Restriction Extends Chronological Lifespan of Budding Yeast by Avoiding Commonly Used Auxotrophic Marker Nutrient Starvation. Yeast.

[193] Lewis A.G., Carmichael L., Wang R.Y., Gibney P.A. (2024). Characterizing a panel of amino acid auxotrophs under auxotrophic starvation conditions. Yeast.

[194] Shenhar B., Pridham G., De Oliveira T.L., Raz N., Yang Y., Deelen J., Hägg S., Alon U. (2026). Heritability of intrinsic human life span is about 50% when confounding factors are addressed. Science.

[195] Fontana L., Klein S. (2007). Aging, adiposity, and calorie restriction. JAMA.

[196] Hursting S.D., Lavigne J.A., Berrigan D., Perkins S.N., Barrett J.C. (2003). Calorie restriction, aging, and cancer prevention: Mechanisms of action and applicability to humans. Annu Rev. Med..

[197] Bielas J., Herbst A., Widjaja K., Hui J., Aiken J.M., McKenzie D., Miller R.A., Brooks S.V., Wanagat J. (2018). Long term rapamycin treatment improves mitochondrial DNA quality in aging mice. Exp. Gerontol..

[198] Selvarani R., Mohammed S., Richardson A. (2021). Effect of rapamycin on aging and age-related diseases-past and future. Geroscience.

[199] Torrent C., Gagliardi C., Fülle N., Antignano I., Bernis M.E., Stork M., Bano D., Capasso M., Keane L. (2026). Long-term rapamycin treatment suppresses IL-17-producing gamma delta T cells and blunts neuroinflammation in aging. PLoS ONE.

[200] Lee M.B., Hill C.M., Bitto A., Kaeberlein M. (2021). Antiaging diets: Separating fact from fiction. Science.

[201] Houde V.P., Brûlé S., Festuccia W.T., Blanchard P.G., Bellmann K., Deshaies Y., Marette A. (2010). Chronic rapamycin treatment causes glucose intolerance and hyperlipidemia by upregulating hepatic gluconeogenesis and impairing lipid deposition in adipose tissue. Diabetes.

[202] Kirby D., Waszak C., Ziegler J. (1991). Six school-based clinics: Their reproductive health services and impact on sexual behavior. Fam. Plan. Perspect..

[203] Oliveira P.F., Cheng C.Y., Alves M.G. (2017). Emerging Role for Mammalian Target of Rapamycin in Male Fertility. Trends Endocrinol. Metab..

[204] Lee D.J.W., Hodzic Kuerec A., Maier A.B. (2024). Targeting ageing with rapamycin and its derivatives in humans: A systematic review. Lancet Healthy Longev..

[205] Kolbe D., Dose J., Putter P., Ziemann M., Laudes M., Slagboom P.E., Franke A., Deelen J., Nebel A., Center R.G. (2025). German longevity study reveals novel rare pro-longevity alleles clustering in mTOR signaling pathway. Geroscience.

[206] van Heemst D. (2010). Insulin, IGF-1 and longevity. Aging Dis..

[207] Willcox B.J., Donlon T.A., He Q., Chen R., Grove J.S., Yano K., Masaki K.H., Willcox D.C., Rodriguez B., Curb J.D. (2008). FOXO3A genotype is strongly associated with human longevity. Proc. Natl. Acad. Sci. USA.

[208] Li N., Luo H., Liu X., Ma S., Lin H., Chen R., Hao F., Zhang D. (2016). Association study of polymorphisms in FOXO3, AKT1 and IGF-2R genes with human longevity in a Han Chinese population. Oncotarget.

[209] Lee S., Dong H.H. (2017). FoxO integration of insulin signaling with glucose and lipid metabolism. J. Endocrinol.

[210] Sanese P., Forte G., Disciglio V., Grossi V., Simone C. (2019). FOXO3 on the Road to Longevity: Lessons From SNPs and Chromatin Hubs. Comput Struct. Biotechnol. J..

[211] Kenyon C.J. (2010). The genetics of ageing. Nature.

[212] Lyon A., Agius T., Macarthur M.R., Kiesworo K., Stavart L., Allagnat F., Mitchell S.J., Riella L.V., Uygun K., Yeh H. (2024). Dietary or pharmacological inhibition of insulin-like growth factor-1 protects from renal ischemia-reperfusion injury in mice. iScience.

[213] Essaghir A., Dif N., Marbehant C.Y., Coffer P.J., Demoulin J.B. (2009). The transcription of FOXO genes is stimulated by FOXO3 and repressed by growth factors. J. Biol. Chem..

[214] Du S., Zheng H. (2021). Role of FoxO transcription factors in aging and age-related metabolic and neurodegenerative diseases. Cell Biosci..

[215] Shimazu T., Hirschey M.D., Newman J., He W., Shirakawa K., Le Moan N., Grueter C.A., Lim H., Saunders L.R., Stevens R.D. (2013). Suppression of oxidative stress by β-hydroxybutyrate, an endogenous histone deacetylase inhibitor. Science.

[216] Webb A.E., Brunet A. (2014). FOXO transcription factors: Key regulators of cellular quality control. Trends Biochem. Sci..

[217] Anand G.S., Hotchko M., Brown S.H., Ten Eyck L.F., Komives E.A., Taylor S.S. (2007). R-subunit isoform specificity in protein kinase A: Distinct features of protein interfaces in PKA types I and II by amide H/2H exchange mass spectrometry. J. Mol. Biol..

[218] Enns L.C., Pettan-Brewer C., Ladiges W. (2010). Protein kinase A is a target for aging and the aging heart. Aging.

[219] Cheng C.W., Adams G.B., Perin L., Wei M., Zhou X., Lam B.S., Da Sacco S., Mirisola M., Quinn D.I., Dorff T.B. (2014). Prolonged fasting reduces IGF-1/PKA to promote hematopoietic-stem-cell-based regeneration and reverse immunosuppression. Cell Stem Cell.

[220] Timmers P.R.H.J., Wilson J.F., Joshi P.K., Deelen J. (2020). Multivariate genomic scan implicates novel loci and haem metabolism in human ageing. Nat. Commun..

[221] Abondio P., Sazzini M., Garagnani P., Boattini A., Monti D., Franceschi C., Luiselli D., Giuliani C. (2019). The Genetic Variability of APOE in Different Human Populations and Its Implications for Longevity. Genes.

[222] Broer L., Buchman A.S., Deelen J., Evans D.S., Faul J.D., Lunetta K.L., Sebastiani P., Smith J.A., Smith A.V., Tanaka T. (2015). GWAS of longevity in CHARGE consortium confirms APOE and FOXO3 candidacy. J. Gerontol. A Biol. Sci. Med. Sci..

[223] Gerdes L.U., Jeune B., Ranberg K.A., Nybo H., Vaupel J.W. (2000). Estimation of apolipoprotein E genotype-specific relative mortality risks from the distribution of genotypes in centenarians and middle-aged men: Apolipoprotein E gene is a “frailty gene,” not a “longevity gene”. Genet. Epidemiol..

[224] Bin-Jumah M.N., Nadeem M.S., Gilani S.J., Al-Abbasi F.A., Ullah I., Alzarea S.I., Ghoneim M.M., Alshehri S., Uddin A., Murtaza B.N. (2022). Genes and Longevity of Lifespan. Int. J. Mol. Sci..

[225] Hook M., Roy S., Williams E.G., Bou Sleiman M., Mozhui K., Nelson J.F., Lu L., Auwerx J., Williams R.W. (2018). Genetic cartography of longevity in humans and mice: Current landscape and horizons. Biochim. Biophys. Acta Mol. Basis Dis..

[226] Shadyab A.H., LaCroix A.Z. (2015). Genetic factors associated with longevity: A review of recent findings. Ageing Res. Rev..

[227] Murabito J.M., Yuan R., Lunetta K.L. (2012). The search for longevity and healthy aging genes: Insights from epidemiological studies and samples of long-lived individuals. J. Gerontol. A Biol. Sci. Med. Sci..

[228] Li Y., Tian X., Luo J., Bao T., Wang S., Wu X. (2024). Molecular mechanisms of aging and anti-aging strategies. Cell Commun. Signal..

[229] Santos-Lozano A., Santamarina A., Pareja-Galeano H., Sanchis-Gomar F., Fiuza-Luces C., Cristi-Montero C., Bernal-Pino A., Lucia A., Garatachea N. (2016). The genetics of exceptional longevity: Insights from centenarians. Maturitas.

[230] Franzke B., Neubauer O., Wagner K.H. (2015). Super DNAging-New insights into DNA integrity, genome stability and telomeres in the oldest old. Mutat. Res. Rev. Mutat. Res..

[231] Furman D., Campisi J., Verdin E., Carrera-Bastos P., Targ S., Franceschi C., Ferrucci L., Gilroy D.W., Fasano A., Miller G.W. (2019). Chronic inflammation in the etiology of disease across the life span. Nat. Med..

